# Supplementary material for: Principled distillation of UK Biobank phenotype data reveals underlying structure in human variation
Source: Nat Hum Behav. 2024 Jul 4;8(8):1599–615. doi: 10.1038/s41562-024-01909-5 (PMC11343713; doi:10.1038/s41562-024-01909-5)
Supplement: Supplementary file 1 — Supplementary Text and Figs. 1–11. [file 41562_2024_1909_MOESM1_ESM.pdf]

nature human behaviour

Supplementary information https://doi.org/10.1038/s41562-024-01909-5

Principled distillation of UK Biobank phenotype data reveals underlying structure in human variation

In the format provided by the authors and unedited

Table of Contents

Selection and composition of core data group 2
  Selection of individuals 2
  Selection of items 4
  Characteristics of final core data group 5
Selection of items to omit for collinearity 7
Exploratory factor analysis 9
PCA vs. EFA comparison 11
  Methodological comparison 12
  Results 14
Confirmatory factor analysis 16
  Selection of minimum loading for factor inclusion 17
  Multiple imputation of core data group 18
  Computational implementation of structural equation modeling 20
  Iterative model refinement 21
  Constrained confirmatory factor analysis 23
  Assessment of model fit 23
Differences between EFA and CFA 25
Factor score generation 27
  Bartlett estimator 29
  Thomson-Thurstone estimator 31
  Modifications for categorical and missing data 33
    Measurement scale for categorical variables 33
    Transformed loadings 34
    Transformed residual variances 36
    Estimating factor scores with missingness 38
    Heteroskedasticity of Bartlett factor scores by missingness pattern 40
    Missingness-based heteroskedasticity and bias for Thomson-Thurstone scores 42
    Minimum correlation with complete data scores 45
  Validation of factor scoring methods in the core data group 47
Notes on modeling and limitations 49
  Impact of input items on factor structure 50
  Impact of questionnaire structure 51
  Extreme outliers on continuous variables 53
  Modeling of non-continuous items 54
  Forced orthogonality between factors 54
  Selection of “nuisance” covariates 56
  Assumptions of factor invariance 57
  Impact of structured missingness 57
Latent causal variable analysis 59
Tissue specificity of shared and nonshared genetic effects 60
  Methods 61
  Results 62
Genetic correlations between prior SES GWAS and all factors 64
References 65
Supplementary Figures 70

Selection and composition of core data group

Selection of individuals

Factor analysis relies on the formation of a stable correlation matrix as input. However, the UK Biobank was not designed as a single survey measure. It instead consists of numerous self-report surveys, interviews, and assessments given across multiple timepoints[1]. Missingness is introduced at the assessment level through selective response behaviors on the part of participants (e.g., electing to respond to a voluntary follow-up questionnaire), selective ascertainment (e.g., questions only asked of self-identified females or smokers), and later introduction into the UKB battery. The structured missingness introduced by this differential completeness is problematic for factor analysis, which relies upon the correlation matrix being consistent across individuals in the study population. This assumption is less likely to hold when pairwise elements of the correlation matrix are estimated from substantively different sets of individuals. To ensure sufficient pairwise overlap between individuals responding to different surveys and assessments in our core data group, we first considered inter- and intra-survey missingness, identifying a group of individuals who had usable phenotypic data across a wide range of assessments.

We first identified common patterns of structured missingness across individuals in UKB. Starting with the 2,772 phenotypes for which GWAS were performed across both sexes in the Neale Lab UKB Round 2 mega-GWAS (https://www.nealelab.is/uk-biobank/ukbround2announcement), those outcomes derived from a single question were collapsed into one item for the purposes of evaluating missingness patterns (e.g., in the

case of categorical-multiple items, in which a participant could choose any number of responses from a list). We then assigned items to their most specific category based on the UKB tree structure (https://biobank.ctsu.ox.ac.uk/crystal/browse.cgi). To examine patterns of missingness, we then generated a null correlation matrix for all items. Within each category, all items with missingness correlations r>0.95 were merged. These collections of items with highly similar missingness patterns within each predefined category are called "questionnaires" in our analyses.

Of all the questionnaires, those that were asked only of a specific demographic subgroup of individuals (e.g., male- or female-specific questionnaires) or for which inclusion was conditional on a specific index event (e.g., cancer and death registries or the maternity questionnaire) were dropped. We then considered questionnaires with sample sizes of between 75,000 and 250,000; those with sample sizes of less than 75,000 would severely restrict the size of our core data group, while those with sample sizes greater than 250,000 were unlikely to require special consideration for missingness. Next, we dropped questionnaires with less than 5 items. This left us with 6 questionnaires: 1) the claudication and peripheral artery disease questionnaire from the touchscreen survey; 2) the mental health questionnaire from the online follow-up; 3) the work environment questionnaire from the online follow-up; 4) cognitive function measures; 5) physical measures (mostly: blood pressure measures, heel measures, and a hearing test); and 6) eye and vision measures.

Finally, we selected individuals who were missing at most 1 of the 6 questionnaires (N=42,325). This is done to ensure adequate overlap of individuals across questionnaires

and also to help better ensure that questionnaire-level missingness was reasonably occurring at random.

Selection of items

After identifying a group of 42,325 individuals with sufficient completion of less commonly answered questionnaires, we then identified items for which pairwise correlations could be reasonably estimated, and for which missingness was reasonably occurring at random (e.g., was not dependent on an individual's would-be response value). In doing so, we eliminated items for which a response was dependent upon a response to a previous item, items with low prevalence, and items with relatively high missingness, even within the analytic core group of individuals.

Starting with the 2,772 items, we first removed those that were asked only of a specific demographic subgroup of individuals (e.g., male- or female-specific questionnaires) or for which inclusion was conditional on a specific index event (e.g., cancer and death registries; 113 items). We next removed all items with N<30,000 in our core data group (566 items), and, in the case of binary items, those with a prevalence of <1% in either the core data group or full EUR dataset (1,182 items).

We then formed a cross-item Pearson correlation matrix and identified pairs of items for which a correlation could not be estimated, likely indicating dependencies in missingness across the items. We sorted items by the number of "NaN" values they shared with other items, and we excluded the less prevalent item for any "gatekeeper" items or items that were dependent on other items. For example, since the vast majority of the sample reported having drank alcohol (core group: 97.6%; full group: 96.9%), we excluded the

"alcohol drinker status" item for which only a small proportion of individuals reported not drinking, in favor of keeping the downstream questions on weekly alcohol intake. Conversely, since the vast majority of the sample was not adopted (core group: 98.8%; full group: 98.6%), we included the “adopted as a child” item and did not include the downstream questions about adoption. Resolution of these dependencies (removed 13 items) resulted in a final item count of 898. Below we provide more information about demographic characteristics and item composition for our core data group.

Characteristics of final core data group

The core data group was comprised of 42,325 individuals with a high rate of questionnaire completion, along with (1) 898 items with a high rate of completion among those individuals; (2) sufficient prevalence for binary variables (>1%) among both the core and full EUR data groups; and (3) no completely dependent inter-item missingness. The overall missingness rate was 9.1%, with missingness on each item up to 28.6% (SD: 10.7%), and for each individual up to 33.3% (SD: 7.9%). For individuals not within the core data group, item- and individual-level missingness was substantially higher, at 33.4% (SDs: 37.5% and 9.9%, respectively). Individuals in the core data group represented 10 of the 22 UKB assessment centers: Sheffield (N=8,155), Croydon (N=7,919), Hounslow (N=7,618), Birmingham (N=6,590), Liverpool (N=5,229), Middlesborough (N=3,071), Bristol (N=2,338), Nottingham (N=915), Swansea (N=398), and Wrexham (N=92).

Completion rates within the core data group for the 6 questionnaires on which the individuals were selected were as follows: 99.7% (N=42,205) for the claudication and peripheral artery disease questionnaire from the touchscreen survey; 89.8% (N=38,001)

for the mental health questionnaire from the online follow-up; 74.7% (N=31,620) for the work environment questionnaire from the online follow-up; 100% (N=42,318) for the cognitive function measures; 100% (N=42,325) for the physical measures; and 79.8% (N=33,787) for eye and vision measures. A total of 18,631 individuals (44.0% of the core data group) completed all 6 questionnaires.

The causes of structured missingness for these different questionnaires differed: both the mental health and work environment questionnaires were online follow-ups to the original assessment, and participation was contingent on both providing a valid email address and opting in to these later assessments. In contrast, the 4 other questionnaires were added to the initial assessment later during recruitment, such that certain centers that completed recruitment earlier did not administer these questionnaires to any or all of their participants. Within the core data group, this is evident in the eye and vision measures questionnaire, which was not completed by anyone at 4 of the 10 assessment centers (i.e., Bristol, Nottingham, Middlesborough, and Wrexham), and by only 26.9% of individuals at the Swansea assessment center.

A plurality of overall items were taken from the initial touchscreen questionnaire (343 items), with a substantial number taken from the online follow-up as well (239 items, though these contained 159 employment history questions that included both raw and derived job codes). See **Supplementary Table 1** for a full breakdown of items by category.

Individuals within the core data group were equivalent in age to those not included in the core data group (M=56.8 for both; t=0.556, p=0.578), but more were female (55.6% core

group, 53.5% non-core group; χ^2=66.412, p<0.001) and substantially more likely to report having completed college or university (45.7% core group, 30.7% non-core group; χ^2=3816.0, p<0.001). These demographic shifts are consistent with prior literature demonstrating higher response and completion rates for females and individuals with higher educational attainment [2–4].

For our GWAS analyses, because of the differences between the core and full groups and because the factor analysis was modeled and tested exclusively on the core data group, we performed GWAS in both groups to allow for potential comparisons in genetic architecture between them. Though the main results presented in the paper are from the full EUR group, heritability estimates across the groups were generally concordant (range absolute difference: 0.001-0.051, mean=0.013(0.012)), and genetic correlations ranged from all high to very high (range: 0.864-1.101, mean=0.990(0.057); **Supplementary Figure 1**).

Selection of items to omit for collinearity

Though we identified a group of individuals with low *structured* missingness at the survey level, and a corresponding group of items with low missingness among this group, sufficient prevalence (>1%), and lack of cross-item missingness dependencies, additional adjustments needed to be made to facilitate the factor analysis algorithm. Specifically, issues of pairwise collinearity and multicollinearity needed to be resolved.

To address multicollinearity (e.g., perfect prediction of a variable by a combination of other variables), we first made sure that no item clusters existed for which both derived and component items were included. For example, items from a neuroticism questionnaire

were originally included alongside a summed composite score; in this case, we removed the score in favor of keeping the items. Conversely, raw job codes were included alongside derived job codes, which combined multiple raw codes into larger "umbrella" categories. In this case, we kept the derived job codes, as prevalences for these items were increased relative to raw codes. A total of 67 items were removed from such item clusters.

We next removed items for which >50% of variance, once residualizing for covariates, was dependent upon the missingness of one or more items, likely reflective of structured missingness (6 items). This is similar to the above removals for inestimable correlations due to dependent missingness. For example, an item indicating whether or not a hearing test was performed was removed, as its variance was heavily dependent upon the missingness patterns of hearing test outcomes.

Next, starting with a Pearson correlation matrix residualized for our chosen "nuisance" covariates (i.e., first 20 genetic PCs, age, chromosomal sex, age^2, age-x-chromosomal sex, and age^2-x-chromosomal sex) in the modelling subset of the core data group (N=33,860), we identified all pairwise correlations r>0.95. For each of these pairs, one variable was removed (43 items); we prioritized keeping the variable with the least missingness in the core dataset.

We next identified items for which the squared multiple correlation (SMC) was >0.98. Most of these items had obvious reasons for near perfect prediction; for example, some phenotypes were derived from a single question in which a participant could select only one answer. One phenotype from each cluster of items was removed; in most cases we

selected the phenotype with the lowest prevalence or least amount of variance (10 items). Three multicollinear item clusters remained for which survey response patterns were obviously not the cause: eye measures, body fat measures, and blood assays. A clinician was consulted in these cases, and items were removed until perfect prediction (SMC>0.98) stopped (33 items).

Finally, we identified 6 additional items that were “None of the above” responses to questions about otherwise unrelated illnesses of family members (e.g., Parkinson’s disease and depression). Given that such items could introduce multicollinearity and were not otherwise informative/meaningful, these items were removed from analyses. Resolution of all collinearity issues resulted in a final item count of 730 to be carried forward into the exploratory factor analysis.

Exploratory factor analysis

We then performed an exploratory factor analysis (EFA) in the modelling subset of the core data group (N=33,860) using these remaining 730 items in order to determine the structure of the data. EFA was performed using the “psych” package[5] in R (version 4.0.2), using the partial Pearson correlation matrix of the 730 items as input.

A number of guidelines exist for the determination of the number of factors to extract within an EFA, though no hard-and-fast rules exist[6,7]. ”Stopping criteria” may be influenced, for example, by raw eigenvalues (e.g., Kaiser’s stopping rule, which suggests extracting the number of factors with eigenvalues >1; [8]), by the number of observed eigenvalues significantly larger than those calculated based on simulated datasets (e.g., as in parallel analysis; [9]), by visual inspection of the scree plot (e.g., selecting a number

of factors around the “elbow” or inflection point, of the line plot of eigenvalues; [10]), by setting a threshold for cumulative variance explained by the factors, or by examining the structural characteristics of the resulting factor solution (e.g., including only the number of “non-trivial” factors).

Eigenvalue-based approaches within our analysis yielded inconsistent results. The scree plot, for example, suggested 30 – 50 factors (**Supplementary Figure 2**). Parallel analysis suggested 177 factors and 253 eigenvalues of the correlation matrix were >1. The latter results are perhaps unsurprising, given the “long tail” of correlations likely to exist across scattered pairs of items across the 730 input items. Given that one of our main goals was to understand larger-scale structure across the phenotypes assessed within UKB, we therefore placed greater emphasis on the inflection point of the scree plot when deciding on the number of factors to extract.

To further refine the number of factors to extract, we devised a custom approach, with the goal to find a stable structural solution with no non-trivial (e.g., <3 items with loadings >0.3) factors. We explored factor solutions using the following factor extraction methods: WLS (weighted least square), GLS (generalized weighted least square), MINRES (minimum residual) and ULS (unweighted least square). In all steps the “varimax” rotation was used to find solutions with orthogonal factors. For each method the number of factors extracted was increased until the occurrence of Heywood and ultra-Heywood cases. Heywood (and ultra-Heywood) cases indicate that common factors explain 100% (or more) of an individual item’s total variance, suggesting that the number of extracted factors is too large. This provided an upper limit for the maximum number of factors for each method: 169 for GLS, 186 for WLS, 38 for ULS and 38 for MINRES. The MINRES

and ULS methods produced almost identical results and so we only used GLS, WLS and MINRES for further analyses.

For each factor extraction method, increasing the number of factors increased the variance explained by the model but at the same time produced “trivial” factors with one or no items having significant loading (magnitude > 0.3). **Supplementary Figure 3** shows the distribution of the number of significant items in the factors for the different models. Because the 169-factor GLS solution and the 186-factor WLS solution produced many factors with only one significant item, we decided to pursue the 38-factor MINRES model. We were also encouraged by the fact that the number of non-empty factors extracted within this model was within the 30-50 factors suggested by visual inspection of the scree plot.

The 38-factor MINRES solution contained one “empty” factor, i.e., one factor with no item loading with magnitude > 0.3. A 37-factor MINRES solution also contained an empty factor. Reducing the number of factors again resulted in a 36-factor MINRES solution (MINRES-36, RMSR = 0.02, variance explained = 18.5%) with no empty nor trivial factors (**Supplementary Figure 3D**).

PCA vs. EFA comparison

Principal components analysis (PCA) and exploratory factor analysis (EFA) are both commonly used dimensionality reduction techniques. Much has been written about the relationship between these methods (e.g. [11–13]). We summarize some key features here, before demonstrating the relationship of their results when applied to the current dataset.

Methodological comparison

First, EFA and PCA have different goals. EFA states a model that the observed correlation between items can be explained by the contribution of a set of unobserved, latent factors and residual measurement error, and then attempts to find loadings that best fit this model to the data, generally according to some fit statistic (e.g. “minres”; [14]). PCA aims to find linear combinations of items that can explain the most variance in the dataset and are uncorrelated from each other, which corresponds to eigenvectors of the correlation matrix (assuming standardization of the variables prior to analysis).

The distinction between these approaches is evident in the output. PCA produces loadings that are weights for linear combination of the items that compute PCs. The comparable values from EFA are factor scoring coefficients which are used to estimate factor scores (with error) from a linear combination of the items, but these coefficients are a post hoc transformation of the factor loadings fitted by the EFA model. Ths transformation exists because the loadings estimate the effect of factors on the items, while PCA calculates a contribution of the item to the PC, i.e. in the opposite direction. Both methods ultimately capitalize on the same correlation structure within the data, e.g. since summing correlated variables will yield higher variance for PCA, but these different starting points lead to somewhat different solutions.

The clearest connection between these two methods exists in “principal axes” factor analysis. In that approach, the correlation matrix is modified to replace the diagonal elements with the squared multiple correlation of prediction of the given item by all other items, and then PCA is performed on the modified matrix to estimate factor loadings. The diagonal elements of the correlations matrix are then updated to the “communalities”

based on the initial fit, i.e. the proportion of the item explained by the fitted factor structure, and PCA is refit. This process is repeated iteratively until convergence. This approach highlights the impact of the factor analysis model differentiating between the structure explained by factors and residual error as compared to PCA run on a standard correlation matrix. In short, PCA, does not distinguish between unique, or error, variance, and common, shared variance.

Second, the model-based approach of EFA enables inference on the fitted model, both for overall model fit and for individual parameters. Inferential procedures for PCA are much less common. Adding distributional assumptions to PCA permits testing the number of components with Tracy-Widom statistics[15] or Bayesian models[16], and a number of related rules of thumb have also been proposed[13], but inference on overall model fit or individual loading is generally not applicable. Being able to assess model fit in factor analysis is useful both for testing our model construction and for evaluating its generalizability in a holdout sample.

Lastly, factor analysis not only extracts a set of factors but also rotates their axes to aid in interpretability. These rotations enforce a "simple structure" on the factor solution by adding additional optimization criteria that resolve the rotational indeterminacy of the multidimensional set of factors. In general these optimization criteria (e.g. "varimax"; [17]) prioritize solutions where each factor has relatively few items with strong loadings, and each item loads strongly on few factors. This ensures a relatively simple structure along each latent axis, generally allowing the nature of each latent factor to be inferred from its top items. In theory this kind of rotation could also be applied to PCA results, but this is

not conventionally part of PCA analysis and lacks the depth of previous literature evaluating the use of rotation in factor analysis.

In sum, while both factor analysis and PCA provide dimensionality reduction that captures the correlation structure observed in phenotypic data, factor analysis has the benefit of providing a testable model and adds a conventional step of factor rotation to identify simpler axes for understanding the identified structure.

Results

To demonstrate how our choice of factor analysis compares to PCA for the purpose of our analyses, we additionally perform PCA on the same partial Pearson correlation matrix that served as input to the EFA.

The first 36 principal components explained 21.6% of the variance in the input phenotypes. This is slightly higher than the variance explained by the EFA solution (18.5%), which is perhaps unsurprising given that the PCA algorithm is "greedy" and seeks to maximize explained variance in the input matrix. By comparison, the EFA algorithm optimizes for the accuracy of approximating the observed correlation between variables rather than directly optimizing the variance explained.

Comparison of the identified factors and PCs shows broad differences. Estimated correlations between factor scores and principal components reveal only sporadic overlap between the contents of the factors and components (**Extended Data Figure 3A**). Inspection of the top items (i.e., by loading for EFA, by scoring coefficient for PCA) reveals that the content of each PC is less clearly differentiated than in the factor solution. A number of components, but not factors, share the same top item; for example, PCs 29

and 30 both have “Diabetes diagnosed by doctor” as their top item. As a result fewer items are strongly represented by the PCs: 175 items (or 24.0%) fall within the top 5 item in one or more EFA factors, but in the PCA this number falls to 105 (14.4%). The top items observed for each PC are also often harder to interpret (**Supplementary Table 3**).

The lack of alignment between the PCs and the EFA factors is consistent with the axes of variation identified in the EFA being rotated based on the “varimax” criterion for simple structure while the PCA axes are unrotated. The majority of the variance in the factor scores can be jointly explained by the PCs (**Extended Data Figure 3B**), suggesting that the overall space extracted by the two methods is similar. The EFA however yields factors that generally have sparser loadings, with loadings from the PCA generally involving moderate contributions from a much longer list of variables (**Extended Data Figure 3C&D**).

Still, for some factors the correspondence to the PCs is sufficiently diffuse that the content of the factor would likely be overlooked by inspection of the PCs alone. For example, while 76.3% of variance in Factor 23 can be explained by the 36 PCs, no single PC explains more than 11.3% of the factor’s variance. Furthermore, the most predictive PC, PC21, include variables reflecting OTC pain medication use and household size, rather than the variables related to physical activity and health behaviors captured by Factor 23. Similarly, only two PCs individually explain >10% of variance in Factor 9, which in the FA contains items related to trauma and its sequelae: PC1 (explaining 11.0%) is composed primarily of depression-related items, while PC29 (explaining 11.7%) is led by items related to hearing.

Overall, EFA and PCA are both common dimensionality reduction methods with valid potential applications to large-scale phenotypic data, but represent different priorities. However, when the desire exists to evaluate underlying structure across a dataset in addition to simply reducing data complexity, factor analysis is better aligned with those goals.

Confirmatory factor analysis

Model refinement was carried out on an imputed version of the core data group (see **Multiple imputation of core data group**) using a confirmatory factor analysis framework in structural equation modeling using lavaan[18] (version 0.6-3) in R as a template, with extensive modifications (see **Computing aspects of structural equation modeling**). A confirmatory factor analysis tests the fit of a predefined model to the data. In contrast to an exploratory factor analysis, paths are constrained such that certain factors influence only certain prespecified observed variables. In the case, we wanted to test the fit of the model as defined by the EFA in the same modeling subset (now N=33,854 due to participant withdrawals during the course of the study) when modeling the covariance structure of all items appropriately and restricting the paths being estimated.

As in the EFA, we modelled all latent factors as orthogonal. Observed variables were assigned to latent factors if their loadings were >0.10 in the EFA (see **Selection of minimum loading for factor inclusion**). Continuous variables were standardized, and all variables were residualized for exogenous nuisance covariates (i.e., first 20 genetic PCs, age, chromosomal sex, age^2, age-x-chromosomal sex, and age^2-x-chromosomal sex). These "nuisance" covariates were treated as fixed parameters in the model.

Variances of all observed and latent variables were fixed to 1. Correlations between variables were estimated as appropriate (e.g., polychoric for pairs of ordered variables, Pearson for pairs of continuous variables, and polyserial for pairs containing one of each), assuming an underlying normal distribution. Model parameters were estimated using diagonal weighted least squares (DWLS), with final robust standard errors and test statistics calculated using a scale-shifted approach (i.e., the WLSMV option in lavaan).

Selection of minimum loading for factor inclusion

To understand the range of meaningful factor loadings, we generated random data and compared the factor loadings for these random items to the loadings of the original 730 items. Three different kinds of random data were used: 1) Random normal ("rnorm" function in R, mean = 0, sd = 1), 100 repetitions; 2) Random binary traits ("rbinom" function in R, 100 repetitions each, p = 0.01, 0.05, 0.1, 0.2, 0.3,0.4, 0.5); 3) Random exponential traits ("rexp" function in R, rate =1, 100 repetitions). These data were corrected for covariates in the same way as the original 730 items, and combined correlation matrices were calculated with the main dataset. Once again 36 factors were extracted from these merged matrices using the MINRES method. In all cases the absolute value of the factor loadings of the random items were less than 0.03 (**Supplementary Figure 4**). Based on these analyses we used a conservative cutoff of loading magnitude > 0.1 when considering items for inclusion for confirmatory factor analysis.

Multiple imputation of core data group

The core group dataset (N=42,325) was imputed using the Multivariate Imputation by Chained Equations (MICE) package[19] in R. Though the overall missingness of the core data group was low for items included in the CFA (9.3%), structural equation modeling of the dataset in lavaan required complete data, and we employed multiple imputation as a principled way to accomplish this complete data requirement.

We imputed all variables carried forward into the model refinement step (564 items; i.e., loadings >0.1 on a factor in the EFA). Of these 564 variables, 531 had missing values in the core dataset. To aid in imputation, we included all covariates, as well as 20 additional auxiliary variables (e.g., previously excluded “gatekeeper” items and assessment center) that could help explain patterns of missingness.

We chose classification and regression trees (CART) as an imputation method after testing this method and three other MICE methods appropriate for use with both ordered and continuous input variables; these included predictive mean matching, linear or logistic regression (as appropriate), and random forest on 104 variables purposefully selected to represent a range of data types and prevalence rates in the modeling subset of the core group. We used 10 iterations each to yield 5 imputations per method. Of the methods tested, CART demonstrated consistent (and relatively efficient) convergence, yielded values within the expected bounds of the observed values, and yielded mean values across imputations that were moderately correlated to the true, masked values (mean r_{MAR}=0.518[0.222]; mean r_{MCAR}=0.524[0.222]) in versions of our dataset with an additional 10% artificially induced missingness in either a missing at random (MAR) or missing completely at random (MCAR) pattern. There was little evidence of systematic bias using

this method (mean standardized difference, MAR=-0.001[0.018]; mean standardized difference, MCAR=-0.001[0.017]). Importantly, across all pairs of variables, the correlations generated using datasets with artificially induced missingness imputed via CART were almost identical to those without such induced missingess for both MAR (e.g., for one of the 5 imputations: mean absolute difference = 0.009[0.011]) and MCAR (mean absolute difference = 0.010[0.012]).

Using the CART algorithm, we further restricted predictors for each item to those with a pairwise r>0.1 for either the target value or missingness pattern of the target item (for items with >50 individuals missing). These correlation matrices were constructed using the psych package's[5] "mixedCor" function in R, with functionality modeled after MICE's "quickpred" function. We additionally restricted predictors to those for which the missingness pattern was not dependent on that of the target item, with such dependence defined as items for which >50% of individuals missing the target item were also missing the predictor item.

We generated 10 imputations of the core data group, with 10 iterations per imputation. Visual inspection of mean values across imputations revealed good convergence for all variables with any missingness. In the context of the CFA, we conducted sensitivity analyses to determine the degree to which imputation versus pairwise deletion may affect our results. Specifically, we compared the correlation matrices within the core, low-missingness sample resulting from pairwise deletion versus imputation and found little difference (e.g., for one imputation vs. the pairwise matrix of correlations: mean absolute difference = 0.001[0.003]; **Supplementary Figure 5**). We viewed this as evidence that our decision to use pairwise deletion in the EFA, though suboptimal, was unlikely to affect

results. Similarly, going forward, given the strong concordance across pairwise nonmissing data and the individual imputations, as well as across the individual imputations themselves (mean of mean differences across all pairwise combinations of imputations: 0.002[1.38e-5]), we decided to proceed with modelling using only a single imputation for relative computational ease, and instead used the multiple imputations to validate our factor scoring algorithm later on.

Computational implementation of structural equation modeling

Structural equation modeling was carried out using lavaan ([18]; version 0.6-3) as a template, with significant modifications made to achieve computational efficiency. It is unsurprising that modifications were necessary for our application, given the conventionally a factor analysis with 120 items (vs. 564 here), 3 factors (vs. 35 here), and 2,400 people (vs. 33,854 in our core data modelling group) would be considered "large" [20]. Full-information maximum likelihood, or "FIML" methods, often viewed as the "gold-standard" for SEM in the context of missingness, proved infeasible for our use case due to the computational burden of requiring high-dimensional numerical integration [21]. DWLS is often suggested as a reasonable, if imperfect, alternative to FIML that is able to appropriately handle missingness and categorical data [22–24]. Nonetheless, DWLS requires the creation of a weight matrix W which contains the asymptotic variance-covariance of each element of the observed covariance matrix [25]. As such, W scales quarticly with increasing numbers of observed items (i.e., in our case, 564), at a heavy computational cost.

Leveraging matrix sparsity (e.g., using R's Matrix package [26], and R's bigstatsr package [27]), an optimized BLAS (i.e., Intel MKL BLAS), explicit parallelization (e.g., using R's doparallel package [28]), and reduction of linear algebra computations based on our specific use case, we were able to greatly reduce the computational and time complexity of the analysis. Adapted code is available via github [LINK TO BE ADDED UPON ACCEPTANCE]. Nonetheless, parts of the computation–notably, the computation of W–required the use of a Google Cloud Compute virtual machine with 80 vCPUs and 1.9TB of RAM (i.e., m1-ultramem-80). Future advances in computation, including the use of GPUs, along with continued methods development [29,30], may allow for FA and SEM to be more widely applied at the biobank scale in analyses such as this one.

Iterative model refinement

Fitting the EFA-derived model using CFA yielded a number of initial errors due to a lack of estimable pairwise correlation (e.g., due to collinearity) and cell sizes of 0 for ordinal variables. One of each pair of collinear items was removed (4 items), and a minimum cell size threshold of 25 within the core data set further resulted in the removal of 5 ordinal variables.

Following these changes, the EFA-derived model (minus the aforementioned variables) yielded 23 Heywood cases (out of 555), characterized by negative residual variance and indicative of overfitting. For the most part, these Heywood cases were related to one of two major issues: 1) pairwise collinearity ($r>0.99$) newly identified due to correct modeling of binary and ordinal variables; 2) multicollinearity due to the inclusion of variables indicating a response of "None of the above" to a certain question. For purposes of

consistency and model stability, we removed all remaining “None of the above” variables (13 items; see **Impact of questionnaire structure**), as well as one of each pair of inestimable or r>0.99 correlations present in either the training or testing subgroup, regardless of whether those items were linked to a Heywood case (12 items).

Additionally, in looking at the remaining Heywood cases, it appeared that some were likely due to pairwise correlations just below the r>0.99 threshold, or unresolved multicollinearity across item clusters (i.e., smoking and traffic items). We removed one of each pair of items with a pairwise correlation just <0.99 that was likely causing a Heywood case (2 items), and removed 9 additional items due to multicollinearity. Finally, we observed that low cell counts across pairs of variables and/or covariates were further causing problems with estimating correlations; we thus removed 3 additional items.

After systematically removing these items (516 items remaining), 7 Heywood cases remained; the majority of these were due to pairwise correlations just below the r>0.99 threshold and item-cluster multicollinearity. Items were iteratively removed until no negative residual variances remained (11 items), leaving 505 items in the final model. Additionally, once these items were removed, one factor (Factor 8) overlapped completely with Factor 4 and was removed to facilitate model fitting (see **Differences between EFA and CFA**). **Supplementary Table 4** documents the reason for each variable’s exclusion from the EFA to the final factor model. Finally, we noticed that misfit in certain parts of the model was being driven by the presence of extreme outliers (see **Extreme outliers of continuous variables**). Therefore, we removed from analysis all individuals in the core group with values greater than 20 standard deviations from the mean on any continuous

variable (N in modelling group = 52; N in holdout group = 13). This resulted in a final N of 33,802 in the modelling subgroup.

Constrained confirmatory factor analysis

To evaluate the applicability of the factor model beyond the training subgroup, we obtained fit metrics in the validation holdout subgroup (N=8,465; N=8,452 after removing continuous-variable outliers) while constraining the model parameters (i.e., factor loadings) to those estimated in the training subgroup.

Assessment of model fit

Traditional assessments of model fit include model chi-square (p-value should not be significant), root mean square error of the approximation (RMSEA; values 0.01, 0.05, and 0.08 indicate excellent, good, and acceptable fit, respectively), standardized root mean squared residual (SRMR; values <0.08 indicate good fit), comparative fit index (CFI; values >0.90 indicate good fit), and Tucker Lewis index (TLI; values >0.90 indicate good fit). We report each of these metrics for the modelling and holdout samples in **Supplementary Table 2** for the sake of completeness. In addition, where applicable we report both the uncorrected and “scaled” versions of these metrics, where the scaled values rely on the adjusted test statistic from fitting the model with DWLS. We caution that neither of these values are fully robust for use with categorical data, but no better alternatives are currently available without computation of the likelihood, which is currently infeasible here [31,32].

However, as our approach differed from more typical applications of factor analysis– chiefly, in that we were analyzing items across the phenome rather than those originating

within a single scale–not all fit metrics and corresponding cutoffs were deemed equally applicable. We argue that in the context of our analyses, the relative fit indices (i.e., CFI and TLI) are less likely to be informative.

First, our goal is to find a useful summary of the structure of the phenotypic data across UKB, rather than to infer the truth of that structure. Therefore, it is less important how our chosen model fits compared to a null baseline (e.g., relative fit) versus how well the model approximates the data (i.e., absolute fit). This also means we're willing to accept some levels of model misfit in the interest of model parsimony. For instance, we choose to have the CFA model include EFA loadings > 0.1, balancing between a more saturated model with all loadings that appeared to potentially be greater than chance (e.g. > 0.03; see Supplementary Text section **Selection of minimum loading for factor inclusion**) or a sparser model using the conventional rule of thumb (loading > 0.3). We could certainly improve model fit by carrying forward weaker loadings, as evident in the better fit of the EFA (CFI=.883, RMSEA=.015) than the CFA (CFI=.818, RMSEA=.027), but that would come at the expense of having a less parsimonious summary. This same motivation supports focusing on approximate fit (e.g., RMSEA) over exact fit (e.g. chi square).

Second, expectations for what constitutes "good" comparative fit values may be particularly ill-suited to our data. We are modeling an unusually large number of items for factor analysis, and it has been shown that for a given level of misspecification, CFI will report worse fit as the number of items increases [33]. We are also modeling a large number of domains, the correlations across items are, on average, quite low. As a result, the baseline null model for the comparative indices has better fit than would be normal for conventional factor analysis data, leaving less room for our factor model to improve on

the baseline. Specifically, our results suggest the null model for comparative fit would be sufficient to achieve RMSEA=0.063 in the CFA holdout data (i.e., based on rearranging the equation TLI = (1-$RMSEA^2$) / $RMSEA_{null}^2$ to solve for $RMSEA_{null}$), which is far less than the RMSEA=0.153 level of misfit that has been recommended as necessary for comparative fit indices to be informative (https://davidakenny.net/cm/fit.htm).

Lastly, there is room for concern about overreliance on fit statistics of any form and their respective common cutoffs. Lai and Green (2016)[31] provide an excellent overview of features that can cause disagreement between relative and absolute fit and the many pitfalls in trying to draw inferences from their discordance. As such, in addition to favoring RMSEA/absolute fit over CFI in our model evaluation, we directly inspected the model-implied vs. actual correlation matrices to identify the sources of remaining residual misfit (and note that the most prominent areas of misfit are due to “categorical-single” items and medication codes–see Supplement section **Impact of questionnaire structure**).

Differences between EFA and CFA

Our exploratory and confirmatory factor analyses differed in several important ways. First, the exploratory factor analysis was performed on a partial pairwise Pearson correlation matrix, whereas the confirmatory factor analysis was performed on an imputed version of the core data set modeling correlations as appropriate (e.g., polychoric for pairs of ordered variables, Pearson for pairs of continuous variables, and polyserial for pairs containing one of each). Second, in an EFA, all paths and cross loadings are modeled; however, in a CFA, paths are pre-specified in accordance with a hypothesized structure (e.g., in our case, factors were said to include all items with a loading of >0.1 in the EFA).

These changes in modeling resulted in dropping 59 items and one factor when moving from the EFA to CFA (see **Supplementary Table 4** for reasons for dropping each item) in order to facilitate model fitting.

Factor 8, the factor that we dropped from analysis, previously contained items relating to air and noise pollution, as well as road traffic. As we began investigating Heywood cases in the initial model within the CFA framework, it became evident that Factor 8 was primarily serving to separate the covariance due to the traffic items from the rest of the items contained in Factor 4, which encompasses items related to population density, pollution, and transportation. Once items within the traffic cluster were removed in the CFA due to collinearity, the remaining items were almost entirely contained within Factor 4. We therefore removed Factor 8 due to redundancy.

Apart from removing Factor 8, structure and interpretation within each factor was generally consistent. Of the remaining 35 factors, correlation between loadings for the EFA vs. CFA were very high (i.e., r>0.9) for 9 factors (i.e., Factors 1, 6, 17, 21, 22, 23, 25, 26, and 36), high (i.e., r=0.7-0.9) for 15 factors (i.e., Factors 2, 3, 4, 5, 7, 9, 11, 15, 16, 19, 28, 27, 29, 31, and 32), moderate (i.e., r=0.5-0.7) for 6 factors (i.e., Factors 10, 12, 18, 20, 33, and 34), low (i.e., r=0.3-0.5) for 2 factors (i.e., Factors 13 and 24), and very low (i.e., r<0.3) for 3 factors (i.e., Factors 14, 30, and 35).

Factors with low and very low loading correlations were primarily affected by removal of top items or highly correlated clusters of items. For example, the top-loading items in Factor 14 in the EFA were a cluster relating to disability assistance (i.e., "Attendance/disability/mobility allowance: None of the above",

“Attendance/disability/mobility allowance: Disability living allowance”, “Attendance/disability/mobility allowance: Blue badge”, and “Current employment status: Unable to work because of sickness or disability”) that were removed due to multicollinearity in the CFA. The resulting factor relates to long-term disability, but loadings within the factor we reorganized such that items relating to pain medication and joint and bone disease were much more prominent. Similarly, a cluster of items within Factor 30 relating to diet (e.g., “Pork intake”, “Beef intake”, and “Never eat eggs, dairy, wheat, sugar: I eat all of the above”) was removed in the CFA due to low cell counts and multicollinearity issues. As a result, a factor that was previously related to poorer dietary habits became related a mix of poor dietary habits (e.g., “Bread type: White” and decreased “Fresh fruit intake”), blood inflammation/infection markers (e.g., “White blood cell (leukocyte) count” and “Platelet count”), and poor health behaviors (e.g., decreased “Leisure/social activities: Sports club or gym” and “Water intake”).

Our multi-stage approach, which was selected to help minimize computational burden and appropriately handle missingness, made it infeasible for us to return to the EFA and generate a new model of the data. Instead, we chose to move forward with the model suggested by the CFA, in spite of these changes to certain specific factors, due to overall acceptable fit to the data.

Factor score generation

Once the factor model was determined, we then computed factor scores for each latent factor for each individual in the full EUR sample, taking into account differential missingness patterns across individuals.

Given the factor analysis model

$$X = F\Lambda' + \epsilon$$

and the resulting fitted parameter estimates from the CFA, it is possible to estimate each person's latent factor scores as a linear combination of the observed items. In other words, we can define a matrix of factor scoring coefficients A such that

$$\hat{F} = XA$$

And thus for each individual i the estimated factor score for factor t is a weighted sum of the items

$$\hat{f}_{i,t} = \sum_{j} a_{j,t} x_{i,j}$$

If we take the factor model as true then the resulting estimates are of an individual's "true" score for the underlying latent construct, otherwise they simply estimate the value that best approximates the observed data for each individual with the low rank approximation of the complete data modelled by the CFA. These estimated factor scores can then be used in subsequent analyses of how the factor score are related to other variables outside the CFA (e.g., genetics, mortality, other diagnoses).

The current analysis uses two sets of factor scores, corresponding to two different estimation methods to compute the factor scoring coefficients A: (1) Bartlett's method[34,35], used when the estimated factor score is the dependent variable in an analysis (e.g. in the GWAS), and (2) the Thomson-Thurstone (Regression) method[12,36], used when the

estimated factor score is the independent variable in an analysis (e.g., in the phenotypic associations).

This use of two different estimated factor scores, where the choice of factor score estimate to use in a given analysis depends on its placement in the model, follows previous recommendations to avoid biased results in factor score regression[37,38]. Briefly, because these factor score estimation methods differ in how they prioritize correlation and covariance of the estimate with the "true" factor score they have different expected bias in the results of regressions that include the estimated factor score. Using factor scores estimated by Bartlett's method as dependent variables and factor scores estimated by the Thurstone-Thomson method as independent variables avoids this bias in both cases. In addition, both estimators have been extremely well studied in the factor analysis literature, and thus provide a familiar foundation for interpreting the factor scores in the current analysis. Below we introduce both the Bartlett and Thomson-Thurstone estimators for factor scores, followed by the modifications we make to each method to account for the presence of missing data and categorical variables in the current analysis.

Bartlett estimator

Bartlett's estimator[39] for individual factor scores is given by

$$\widehat{F_B} = XA_B$$

$$A_B = \Psi^{-1}\Lambda(\Lambda'\Psi^{-1}\Lambda)^{-1}$$

Where: (1) X is the $n \times p$ vector of p observed variables in the factor model for n individuals, standardized and residualized for exogenous covariates, as in the fitted CFA;

(2) A_B is the $p \times t$ matrix of coefficients to estimate the t factors from p items (i.e., the factor scoring matrix); (3) Λ is the $p \times t$ matrix of factor loadings from the fitted CFA; and (4) Ψ is the $p \times p$ diagonal matrix of residual variances from the fitted CFA (i.e., item uniquenesses).

Bartlett's estimator can be motivated as a weighted least squares estimate that minimizes the residual variance of the items in the factor model after weighting for the expected variance due to the fitted item uniquenesses from the model. Specifically, recalling the factor model

$$X = F\Lambda' + \epsilon$$

(assuming all X have been centered), the Bartlett estimator minimizes

$$\sum_j \frac{\epsilon_{ij}^2}{\Psi_j} = (X - F\Lambda')'\Psi^{-1}(X - F\Lambda')$$

For F, which is consistent with weighted least squares with weights Ψ^{-1}. Using the estimated values from the CFA and following standard weighted least squares this yields

$$\hat{F} = X\hat{\Psi}^{-1}\hat{\Lambda}(\hat{\Lambda}'\hat{\Psi}^{-1}\hat{\Lambda})^{-1} = XA$$

One key property of Bartlett estimator is that it produces unbiased estimates of the effect of variables on the (unobserved) true factor score when used as the dependent variable in factor score regression (as long as the model is correctly specified [38]). Letting Z be a $n \times k$ matrix of of observed data on k variables, regressing $\hat{F}$ on these variables by ordinary least squares will estimate regression coefficients

$$\hat{\beta} = (Z'Z)^{-1}Z'\hat{F}$$

$$= (Z'Z)^{-1}Z'XA$$

$$= (Z'Z)^{-1}Z'(F\Lambda' + \epsilon)\,\widehat{\Psi}^{-1}\widehat{\Lambda}(\widehat{\Lambda}'\widehat{\Psi}^{-1}\widehat{\Lambda})^{-1}$$

$$= (Z'Z)^{-1}Z'F\Lambda'\widehat{\Psi}^{-1}\widehat{\Lambda}\left(\widehat{\Lambda}'^{\widehat{\Psi}^{-1}}\widehat{\Lambda}\right)^{-1} + (Z'Z)^{-1}Z'\epsilon\widehat{\Psi}^{-1}\widehat{\Lambda}(\widehat{\Lambda}'\widehat{\Psi}^{-1}\widehat{\Lambda})^{-1}$$

As $\widehat{\Lambda}$ converges to Λ, as expected for a consistent estimator of Λ in CFA, this reduces to

$$\hat{\beta} \approx (Z'Z)^{-1}Z'F + (Z'Z)^{-1}Z'\epsilon\widehat{\Psi}^{-1}\Lambda(\Lambda'\widehat{\Psi}^{-1}\Lambda)^{-1}$$

Then as long as the k regression values are independent of the unique item residuals ϵ, either directly $(E[Z'\epsilon] = 0$ or as a weighted average across the factor $(E[Z'\epsilon\Psi^{-1}A] = 0$, this leaves

$$\hat{\beta} \approx (Z'Z)^{-1}Z'F$$

i.e., the effect that would be estimated from regressing the "true" score F on Z. It is for this reason that we use the Bartlett estimator for factor scores that are the dependent variable in an analysis. We discuss additional implications of this expectation as it relates to our decision to apply a threshold for minimum correlation with complete data factor scores below.

Thomson-Thurstone estimator

The Thomson-Thurstone estimator [12,36] for individual factor scores is given by

$$\widehat{F_{TT}} = XA_{TT}$$

$$A_{TT} = \Sigma^{-1}\Lambda = \Psi^{-1}\Lambda(I + \Lambda'\Psi^{-1}\Lambda)^{-1}$$

Where: (1) X is the $n \times p$ vector of p observed variables in the factor model for n individuals, standardized and residualized for exogenous covariates, as in the fitted CFA; (2) A is the $p \times t$ matrix of coefficients to estimate the t factors from p items (i.e., the factor scoring matrix); (3) Σ^{-1} is the $p \times p$ covariance matrix for the observed items; (4) Λ is the $p \times t$ matrix of factor loadings from the fitted CFA; (5) Ψ is the $p \times p$ diagonal matrix of residual variances from the fitted CFA (i.e., item uniquenesses); and (6) I is a t-dimensional identity matrix. The latter formulation of A follows from a special case of the matrix inversion lemma[40–42] under the CFA model (i.e., $\Sigma = \Lambda\Lambda' + \Psi$) given the factors are uncorrelated with unit variance. We implement the Thomson-Thurstone estimator using this latter formulation.

The Thomson-Thurstone estimator, also sometimes known as the regression method for factor score estimation, is motivated by regression of the "true" factor scores F on the items X. This can't be done directly since factor scores are unobserved, but by noting that the loadings Λ are the expected covariance between F and X under the model it then follows that the desired regression estimate is given by

$$\hat{F} = X([X'X]^{-1}X'F) = X\Sigma^{-1}\Lambda = XA_{TT}$$

It can be shown[43] that the resulting factor score estimates are the best linear prediction, minimizing the trace and determinant of the expected mean squared error (MSE) matrix

$$E[(\hat{F}_{TT} - F)(\hat{F}_{TT} - F)'].$$

Factor score regression with the Thomson-Thurstone estimator yields unbiased estimates only when the factor score is used as the independent variable in regression with an observed dependent variable[38]. This is the converse of the Bartlett estimator,

which provides unbiased factor score regression results when used as a dependent variable. We therefore use the bias-avoiding method of factor score regression[37,38], using the Bartlett estimator for factor score estimates used as dependent variables and the Thomson-Thurstone estimator for factor score estimates used as independent variables. To preserve the proper scaling of the factor scores with these properties we do not standardize the factor score estimates.

Modifications for categorical and missing data

The above framework for the factor score estimators assumes that all of the item data X is observed. Although this is true for observed continuous items in the model, it is not true for the CFA model where either (a) the observed data is categorical and modelled through a link function or (b) the data is unobserved due to missingness. Ideally, these features could be addressed instead by estimating factor scores with maximum likelihood, but that is not currently computationally feasible. We describe here our solutions for addressing the problem of categorical and missing data, followed by a comparison of the performance of our approach to maximum likelihood estimation with imputed data.

Measurement scale for categorical variables

For categorical items, the CFA model assumes a (ordered) probit link function to connect the observed categorical values to a normally distributed continuous latent value. The estimated loadings $\widehat{\Lambda}$ and residual variances $\widehat{\Psi}^{-1}$ reflect effects for this continuous latent value. The factor scoring estimators therefore estimates the factor scoring coefficients A that would be appropriate for estimating scores based on the unobserved latent variable. The observed categorical variable, however, will have different scaling (e.g. binary 0/1

rather than continuous scale relative to a standard normal distribution) and will have a weaker relationship with the factor due to the information lost when discretizing the underlying continuous variability (e.g., the same phenomenon often described for the impact of dichotomizing continuous variables and the distinction between observed and liability scale heritability for binary traits).

We first address the measurement scale by estimating the expected value of the latent continuous variable for each person for each categorical variable based on the fitted probit link. Specifically, let x_j^* be the continuous latent variable corresponding to the observed categorical variable x_j. Then we estimate the expected value of x_j^* according to a truncated normal distribution with mean equal to the individual's predicted value of the linear model from the probit regression with exogenous covariates $\boldsymbol{z}$, unit variance, and upper and lower truncation thresholds set according to the observed $x_{i,j}$ and the in-sample prevalences of the possible responses $\boldsymbol{p}$. We then use the difference between this $E[x_{i,j}^*|x_{i,j}, \boldsymbol{z}, \boldsymbol{p}]$ and the predicted value from the probit regression as our estimate of the desired latent continuous variable residualized for the exogenous covariates. We substitute this estimated residual in place of the observed $x_{i,j}$ for factor scoring, along with transforming the loadings and residual variances used in each estimator to reflect the attenuated signal in these observed items.

Transformed loadings

While we can approximate the latent continuous values for each categorical item, the expected values do not fully recover the variation of the unobserved latent value. To account for the attenuated signal present in these expected residualized values, we adjust

the loadings using estimating factor scores to reflect the expected weaker covariance between the factor and the observed categorical variable. Again, we let

$$x_j^* = \sum_t \hat{\lambda}_{j,t} f_t + \sum_k \gamma_k z_k + e_j^*$$

be the model-implied fit with exogenous covariates z_k for the continuous latent variable x_j^* corresponding to the observed categorical variable x_j. For the CFA, the terms here are standardized such that $var(f_t) = 1$ for each factor, $var(x_j^* | z_k) = 1$, and each f_t is independent of the covariates and all other factors. Thus each $\hat{\lambda}_{j,t}$ is the estimated partial correlation between the factor and the continuous latent x_j^*. For the purposes of factor score estimation, we want to transform this loading to a value that reflects the partial covariance of the factor with the observed residualized categorical item computed above.

To achieve this transformation, we first note that $\hat{\lambda}_{j,t}$ is effectively an estimate of the polyserial partial correlation between the factor and item (or biserial when the categorical variable is binary). Thus by standard expectations for the polyserial correlation [44] we approximate

$$\hat{\lambda}_{j,t} = cor(f_t, x_j^* | z_k)$$

$$\approx cor(f_t, x_j | z_k) \times \frac{\sigma_x}{\sum_{c \epsilon C} h(c)}$$

where σ_x^2 is the variance of the observed categorical variable x_j and $h(c)$ is the density of the standard normal distribution at the threshold for each category c of the categorical x_j under the assumed probit link function. This expectation would hold more directly if we didn't transform the categorical data to residualize on covariates as described. Still, in most cases the impact of the covariates is small enough that the transformed residualized

variables remain approximately categorical and thus should be well approximated by the expectations for a polyserial correlation; we thus adopt this approximation for convenience. The above expression can then be given in terms of the desired partial covariance as

$$\hat{\lambda}_{j,t} \approx \frac{cov\left(f_t, x_j \middle| z_k\right)}{\sqrt{var(f_t)var\left(x_j \middle| z_k\right)}} \times \frac{\sigma_x}{\sum_{c \epsilon C} h(c)}$$

Rearranging and noting $var(f_t) = 1$ results in

$$cov\left(f_t, x_j \middle| z_k\right) \approx \hat{\lambda}_{j,t}\sqrt{var\left(x_j \middle| z_k\right)} \times \frac{\sum_{c \epsilon C} h(c)}{\sigma_x}$$

where $var\left(x_j \middle| z_k\right)$ and σ_x do not cancel due to the difference in conditioning on covariates z_k. In practice, we estimate $var\left(x_j \middle| z_k\right)$ from the residualized categorical data (described above) and we estimate σ_x from the category probabilities implied by the fitted probit model in the CFA. We then use the resulting estimate of $cov\left(f_t, x_j \middle| z_k\right)$ in place of the estimated loadings $\hat{\lambda}_{j,t}$ for categorical items for the purpose of estimating factor scoring coefficients A. Note however that this transformation is only for factor scoring, and does not affect the loadings reported for the CFA.

Transformed residual variances

Consistent with the loadings $\hat{\lambda}_{j,t}$, the residual variances $\widehat{\Psi}^{-1}$ present in both factor score estimator equations are estimated in the CFA assuming a probit link for categorical variables. For the purpose of factor score estimation we similarly transform these values

to be consistent with the expected attenuation of signal on the transformed and residualized categorical variables.

Specifically, for the CFA $\widehat{\Psi}^{-1}$ is a diagonal matrix whose elements are the residual variance of each (latent) item conditional on the factors and covariates. Categorical items are standardized such that $var(x_j^*|z_k) = 1$, therefore the residual is equal to 1 minus the variance explained by the factors $R^2_{j^*,F}$, where j^* denotes that the variance explained is for the latent continuous item x_j^*.

$$\psi_{jj} = 1 - R^2_{j^*,F}$$

As for the loadings, $R^2_{j^*,F}$ is a squared polyserial correlation. There we can approximate [44]

$$\psi_{jj} \approx 1 - \left(R^2_{j,F} \times \frac{\sigma_x}{\left[\sum_{c \epsilon C} h(c)\right]^2}\right)$$

The corresponding residual variance of the observed categorical items conditional on the covariates and the factors (i.e., the desired value for factor scoring with the observed data) can be expressed as

$$\psi_{jj,obs} \approx var(x_j|z_k) \times (1 - R^2_{j,F})$$

As before, this approximation is inexact due to the transformation and residualization of the categorical items as described above, but we anticipate this approximation will perform adequately, especially when covariate effects are small. Rearrangement and substitution with the approximation for ψ_{jj} leads to

$$\psi_{jj,obs} \approx var\left(x_j \middle| z_k\right) \times (1 - \left[1 - \psi_{jj}\right] \frac{\left[\sum_{c\epsilon C} h(c)\right]^2}{\sigma_x^2})$$

where ψ_{jj} is the estimated residual variance in the CFA and the remaining terms are calculated as in the transformation of the loadings. As with the loadings, these estimated residual variances for the observed categorical items are then substituted into $\widehat{\Psi}^{-1}$ for the estimation of factor scoring coefficients A.

Estimating factor scores with missingness

For individuals with missing data some elements of x_i are unobserved. This prevents calculation of the complete factor scores

$$\hat{f}_{i,t} = \sum_j a_{j,t} x_{i,j}$$

Given the high rates of missingness in UK Biobank, there is obvious interest in being able to estimate these factor scores for individuals with missing data. Thankfully the factor score estimators provides a natural way to use the same estimation framework when some items are unobserved.

Recall that the Bartlett estimator derives from WLS of the items in the factor model. Then if M is a set of unobserved items then we could chose to optimize for

$$\sum_{j \notin M} \frac{\epsilon_{ij}^2}{\Psi_j} = (X_{-M} - F\Lambda'_{-M})'\Psi^{-1}{}_{-M}(X_{-M} - F\Lambda'_{-M})$$

where X_{-M}, Λ'_{-M}, and Ψ_{-M} denote the observed data, loadings, and residual variance matrices omitting the rows and columns corresponding to the unobserved items in M.

This is equivalent to giving zero weight to the amount of residual error in unobserved items. Conceptually, this is the same treatment given to items not present in the CFA that might also reflect the fitted factors. Following the same derivations for the Bartlett estimator, the resulting factor scoring coefficients would be estimated as

$$A_{-M} = \widehat{\Psi}_{-M}^{-1}\widehat{\Lambda}_{-M}(\widehat{\Lambda}'_{-M}\widehat{\Psi}_{-M}^{-1}\widehat{\Lambda}_{-M})^{-1}$$

Thus factor scoring coefficients, and the resulting estimated factor scores $\hat{F} = X_{-M}A_{-M}$, can be calculated for each individual based on their set of available observed items. The only exception is where $\widehat{\Lambda}'_{-M}\widehat{\Psi}_{-M}^{-1}\widehat{\Lambda}_{-M}$ is singular, which will occur when one or more factor have no observed items with non-zero items (i.e., one or more columns of $\widehat{\Lambda}_{-M}$ contains all zeros); in that case we drop the individual from the analysis.

The same argument similarly applies to the Thomson-Thurstone factor scores intended for use as independent variables. Recalling that the Thomson-Thurstone estimator is based on the idea of regressing the "true" factor scores on the available items, subsetting to observed items gives

$$\widehat{F_{-M}} = X_{-M}([X'_{-M}X_{-M}]^{-1}X'_{-M}F) = X_{-M}\hat{\Sigma}_{-\mathrm{M}}^{-1}\widehat{\Lambda}_{-\mathrm{M}} = XA_{TT,-M}$$

Thus as with the Bartlett scores, we can compute Thomson-Thurstone scores for each individual based on the subset of observed items. Individuals missing all items for one or more factors are similarly omitted.

Based on this solution for factor scoring in the presence of missingness, we may have a few concerns. First, in order to maintain constant covariance with other variables the factor scores will be highly heteroskedastic dependent on the missingness pattern.

Second, covariance of other variables with the factor scores will not remain constant if those variables are related to item uniquenesses, potentially influencing regression results and their interpretation at different levels of missingness. Third, the fitted CFA may not have measurement invariance across different missingness levels, and thus the CFA may be misspecified for some missingness patterns.

Heteroskedasticity of Bartlett factor scores by missingness pattern

Because the set of missing variables M varies between individuals, the estimated Bartlett factor scores used as dependent variables will be heteroskedastic as discussed above. When the factor score is used as a dependent variable, we can potentially account for that heteroskedasticity in downstream analyses by estimating the residual variance in the estimated factor score for a given individual as a function of the missingness pattern.

Specifically, we focus on the use case of GWAS of the estimated factor score as the example primary analysis of interest with the factor score as the dependent variable.

$$\hat{f}_{i,t} = \beta_{0,t} + \beta_{1,j,t} SNP_{i,j} + \sum_k \beta_{k,t} z_{i,k} + e_{i,t}$$

i.e., regression of the factor score on a SNP and accompanying GWAS covariates z. Heteroskedasticity will exist here if the residual variance differs between individuals.

$$var(e_{i,t}) = \sigma_{i,t}^2$$

One conventional solution to efficient estimation of regression in the presence of heteroskedasticity is to use weighted least squares (WLS) with weights for each observation proportional to residual variance. Specifically, WLS estimation of this GWAS

model will be optimal if the model is correctly specified and we can compute weights equal to the inverse residual variance, $w_{i,t} = {}^{1}/_{var(e_{i,t})}$.

With this approach it becomes critically valuable that the Bartlett estimator provides unbiased estimates and maintains the covariance of the estimate with the "true" factor score. This ensures that for regression analyses using the factor score as a dependent variable the population coefficients β will remain constant in expectation across groups of individuals regardless of their missingness pattern in estimating $\hat{f}_{i,t}$, as long as the SNP is associated with the factor and not the uniquenesses of the items in the individual factor.

To estimate the expected residual variance, we first note that we expect most SNPs to have small or null effects. Similarly, since the factor scores are constructed from items that have already been residualized on the standard GWAS covariates, we expect little to no covariate effect. Thus we can approximate

$$var(e_{i,t}) = var(\hat{f}_{i,t} | SNP_i, z_i) \approx var(\hat{f}_{i,t})$$

For an individual missing observations for a set of items M, the expected variance of the estimated factor score is

$$var(\hat{f}_{i,t}) = var(X_{-M} A_{-M,t}) = E[A'_{-M,t} X'_{-M} X_{-M} A_{-M,t}]$$

With observed data X_{-M} (with transformation of categorical variables as described previously) and factor scoring matrix A_{-M} computed from the Bartlett estimator for the corresponding missingness pattern. Treating the factor scoring coefficients as fixed,

$$var(\hat{f}_{i,t}) = A'_{-M,t} E[X'_{-M} X_{-M}] A_{-M,t} = A'_{-M,t} S_{-M} A_{-M,t}$$

where S_{-M} is the covariance matrix of the observed items. Assuming that the covariance of the items is constant across the missingness patterns, we can compute S_{-M} as the sample covariance matrix from the pairwise complete observations in X (again, after residualization). Therefore we estimate the weights for WLS as

$$w_{i,t} = \frac{1}{A'_{-M,t} S_{-M} A_{-M,t}}$$

for each individual i with missingness pattern M.

If these WLS weights are correct then the resulting regression will provide the best linear unbiased estimator of the SNP effects in the GWAS. To the extent that these $w_{i,t}$ are incorrect due to estimation error, model misspecification, or other issues, this WLS regression will behave like OLS in the presence of heteroskedasticity: coefficient estimates will remain asymptotically unbiased, but will not be efficiently estimated and their standard errors will tend to be underestimated, increasing type I error rates.

Missingness-based heteroskedasticity and bias for Thomson-Thurstone scores

The variation introduced by differences in missingness patterns is harder to resolve for the independent variable factor scores from the Thomson-Thurstone estimator. First, in order to use WLS as a control for differential information due to missingness when the factor score is an independent variable rather than the dependent variable would require estimation of $var(y_i | \hat{f}_{i,t}, z_i)$, the residual variance of the observed phenotype of interest y conditional on the factor score and covariates. This quantity depends on the true effect of the factor score on y, and unlike the GWAS case it is unlikely that we can assume that

the effect of the factor – as well as the effects of the covariates – are small enough they could be ignored in estimating appropriate WLS weights.

Given the difficulty of estimating appropriate weights for WLS in the independent variable case, we instead address the expected heteroskedasticity from differential missingness in the items used to estimate the Thomson-Thurstone scores using sandwich (i.e., Huber-White [45]) standard errors. Briefly, for linear regression in the presence of heteroskedasticity

$$(X'X)^{-1}X'\widehat{\Omega}X(X'X)^{-1} ,$$

where $\widehat{\Omega}$ is a diagonal matrix of the estimated squared OLS residuals

$$\widehat{\Omega} = diag([y_i - \widehat{y_i}]^2) ,$$

is a consistent estimator of the covariance matrix for sampling error in the regression coefficients. Although this approach does not affect the estimation of the regression coefficients themselves, it does help improve the control of Type I error rates. Additional refinements of the sandwich estimator have been proposed [46], but we opt for the original estimator of White [45] due to its computational efficiency and the minimal impact of further adjustments at large sample sizes [47] like our current analyses in UK Biobank. We use these standard errors for the linear regression analyses (i.e., biomarkers and phecodes). On the other hand, although robust standard errors have been proposed for Cox regression [48–50], they often require auxiliary data or specifying additional likelihoods with modelling assumptions for the error distribution. Therefore for ease of implementation we choose not to apply a heteroskedasticity correction for the current mortality analyses

(though see **Minimum correlation with complete data scores** below which limits the potential differential missingness in the mortality analysis).

In addition to incorrect standard errors, there is also a risk of biased parameter estimates in regression with the independent variable factor scores from the Thomson-Thurstone estimator. As previously noted [38], The Thomson-Thurstone estimator yields factor scores that maintain

$$E\left(y_i \middle| \hat{f}_{i,t,-M}, z_i\right) = \beta_0 + \beta_{1,t}\hat{f}_{i,t,-M} + \sum_k \beta_{k,t} z_{i,k}$$

with the same regression coefficients $\boldsymbol{\beta}$ for any given missingness pattern M in estimation of the factor score. The OLS regression estimator

$$\widehat{\boldsymbol{\beta}} = (X'X)^{-1}X'y$$

however, will not reliably estimate this $\beta_{1,t}$ since under the assumption of homoskedasticity it evaluates the variance of the factor score – as well as its covariance with other covariates – across all individuals in $(X'X)^{-1}$ rather than conditional on missingness. Therefore estimates of $\beta_{1,t}$ will be biased if the factor score has heteroskedasticity and is correlated with other covariates in the model. While corrections for the general case of this “errors in variables” bias have been proposed [51,52], they generally rely on additional instrumental variables or estimating the covariance matrix of estimation error across the covariates, which are not easily available for our factor scores. For our current use cases, we expect the correlation between the factor score and the regression covariates to be generally small, given the factor scores are already estimated

conditional on the exongenous covariates in the CFA model and we do not expect substantial correlation of the factor scores with the added covariates for assessment center, dilution factor for biomarkers, or date of baseline assessment for mortality analyses (indeed differences in the distribution of factor scores by e.g. assessment center would likely imply more general violations of factor invariance assumptions, affecting more than just the phenotypic factor score regressions, see **Notes on modeling and limitations**). This suggests that the bias from using OLS with the Thomson-Thurstone estimator of factor scores as independent variables should be somewhat limited. We therefore do not attempt further adjustments for bias in the independent factor score analyses.

Minimum correlation with complete data scores

Although we can estimate factor scores allowing for missingness, our confidence in those factor scores decreases as the level of missingness increases. For the Bartlett estimator, increased missingness means the factor score is estimated from fewer items, reducing the plausibility of assuming that the predictors of interest (e.g. GWAS SNPs) will be uncorrelated with the sampling variation in the factor score estimate (e.g. that $E[Z'\epsilon] = 0$). For the Thomson-Thurstone estimator, increasing variability in the accuracy of the factor score between individuals due to differential missingness will also exacerbate the regression attenuation caused by the error in the independent variables. For this reason, we choose to limit the impact of missingness on our analyses of the factor scores by restricting analysis to individuals with sufficiently informative factor scores.

Specifically, for each individual we evaluate the expected correlation between the factor score computed with their observed items and their corresponding estimated factor score if all items had been observed (i.e. complete data no missingness).

$$E\left[cor\left(\hat{f}_{i,t}, \hat{f}_{i,t,complete}\right)\right] = E\left[\frac{A'_{-M,t}X'_{-M}XA_t}{\sqrt{A'_{-M,t}X'_{-M}X_{-M}A_{-M,t} \times A'_tX'XA_t}}\right]$$

$$= \frac{A'_{-M,t}S_{-M,\cdot}A_t}{\sqrt{A'_{-M,t}S_{-M,-M}A_{-M,t} \times A'_tSA_t}}$$

where A_t and $A'_{-M,t}$ are the vectors of factor scoring coefficients for factor t with all items and with items observed in missingness pattern M, respectively, and S is the sample covariance matrix for the residualized items, with subscripts denoting subsetting for missingness M on the rows or columns, respectively. Note this expectation assumes that the distribution of the items is independent of the missingness pattern (e.g. that the expectation of $X'_{-M}X$ remains the sample covariance S regardless of M), the same assumption made for WLS weights for the Bartlett estimator. This assumption is likely violated in practice, but we only rely on this assumption here to derive this approximate metric to use for filtering individuals for inclusion in analysis, and do not make any inference on the estimate of $E\left[cor\left(\hat{f}_{i,t}, \hat{f}_{i,t,complete}\right)\right]$.

Based on this metric, we exclude individuals with $E\left[cor\left(\hat{f}_{i,t}, \hat{f}_{i,t,complete}\right)\right]^2 < 0.8$ from analyses with factor scores for factor t. This exclusion was computed and applied for both independent- and dependent-variable use of the factor scores. We chose this threshold based on examination of the distribution of $E\left[cor\left(\hat{f}_{i,t}, \hat{f}_{i,t,complete}\right)\right]$ across individuals for each factor (**Supplementary Figure 6**), with consideration of how this distribution

corresponded to structured missingness for top items and the potential impact of that missingness on item interpretability. We observed this threshold to be more universally liberal in the independent than dependent factor scores, thus we also chose to restrict the sample in independent factor score analyses to only those individuals included in the genetic analyses to allow for better concordance and comparability in samples across phenotypic and genetic analyses.

Validation of factor scoring methods in the core data group

To validate our factor-score-generating methodologies, we compared scores from our methods to those generated using a maximum-likelihood-based (ML) method in lavaan for the core data group. Factor scoring was performed using the ML option in lavaan for all 10 multiple imputations of the core dataset. Though we chose to compare our scoring methods to the ML method in lavaan, the latter cannot be considered the “gold standard,” as numerous factor-scoring methods exist, with each simply relying on and/or prioritizing different assumptions. However, this comparison performed to provide more confidence in our chosen method, which sought to mimic ML estimation in spirit.

To test for phenotypic concordance across methods, we obtained Pearson correlation coefficients between factor scores generated using our method and the mean of those obtained in lavaan across all 10 imputations. These correlation estimates did not weight individuals based on the expected precision of their factor scores, but were restricted to only individuals meeting our analytic inclusion thresholds. Phenotypic correlations were moderate to very high for the dependent-variable (range: 0.531-0.994, mean=0.842(0.137)) and independent-variable (range: 0.540-0.993, mean=0.843[0.132])

formulations. Concordance across dependent- and independent-variable formulations was excellent (range: 0.967-1.000, mean=0.992[0.009]).

We performed GWAS for each factor for the scores generated with lavaan and with our dependent-variable factor scores. GWAS of our scores were conducted using weighted least squares (WLS) regression and appropriate covariates as described in **Methods**. GWAS of the lavaan-generated scores were similarly conducted with WLS and estimated inverse-variance weights $w_{i,t} = 1/var(\hat{f}_{i,t})$. To estimate the variance of lavaan-generated factor scores, we note that under the law of total variance we can decompose

$$Var(\hat{f}_{i,t}) = E\left[Var(\hat{f}_{i,t}|M_i)\right] + Var[E(\hat{f}_{i,t}|M_i)]$$

with the individuals missingness M_i. We then estimate

$$E\left[Var(\hat{f}_{i,t}|M_i)\right] = Var(\hat{f}^{(r)}{}_{i,t})/R$$

as the standard error of the mean across the $R = 10$ imputation replicates $\hat{f}^{(r)}{}_{i,t}$ for individual i, and approximate $Var\left[E(\hat{f}_{i,t}|M_i)\right]$ by the observed variance of the lavaan-generated factor scores across individuals. These weights thus serve as a proxy for imputation quality and missingness proportion across participants analogous to the WLS weights used for the dependent variable factor scores.

For each factor the GWAS results from the two methods were compared using LDSC. Heritability estimates from S-LDSC for each factor were generally concordant across methods (range absolute difference: 0.0002-0.079, mean=0.015(0.019)), with no evidence of systematic bias. The genetic correlation between methods for each factor

was moderate to very high (range: 0.577-1.072, mean=0.976(0.090); **Supplementary Figure 7**).

Lastly, we also compare our use of the Bartlett estimator to the generalized least squares (GLS) approach proposed by Bentler and Yuan[53]. Similar to the WLS approach of the Bartlett estimator, the GLS approach weights using the full covariance matrix of the items Σ rather than the estimated residual variances $\hat{\Psi}$.

$$A_{GLS} = \Sigma^{-1}\Lambda(\Lambda'\Sigma^{-1}\Lambda)^{-1}$$

We find that the GLS estimator using the sample covariance matrix for Σ yields highly similar results to our modified Bartlett estimator despite our modifications to $\hat{\Psi}$ for the Bartlett estimator (results not shown). Along with the comparison to ML estimation, this provides additional reassuring evidence that our chosen factor score estimators are at least somewhat robust to our choice of weighting modifications.

Notes on modeling and limitations

In interpreting the results of this paper, it is important to keep in mind that these factors are not “real” and do not exist as distinct, measurable entities. Instead, they are simply statistical tools that we use to understand relationships between observed variables in this particular cohort and to facilitate downstream analyses by modelling items’ covariance structure with a reduced rank. No prior factor analysis, to our knowledge, has modeled such a large array of variables, across multiple assessments, and spanning multiple data types. Given the extensive adaptations made to traditional factor analysis methodology to use FA as a principled dimensionality reduction technique, we wish to

outline “lessons learned” during the course of the analysis, to inform future studies and also highlight potential limitations. In the following sections, we touch on a host of analytic decisions and assumptions made throughout this project and provide concrete examples of the ways in which they may impact results and limit generalizability.

Impact of input items on factor structure

Factor analysis, as a psychometric technique, is typically used to model inter-item structure within a single questionnaire[10,12,54,55]. Expanding this technique to cover many different questionnaires and assessments types necessitates that the differential *number* of items covering a particular construct will influence the outcome of the factor model. For example, roughly a quarter of all factors (e.g., Factors 5, 13, 17, 19, 24, 26, 27, 32, and 36) contained items derived primarily from a single questionnaire or assessment. Moreover, the top 3 factors identified by the EFA all contained items broadly related to anxiety and depression. This is unsurprising, since of 730 items used in the EFA, 106 were from the mental health questionnaires of either the initial touchscreen (40 items) or online follow-up (66 items). Results would likely look different if other questionnaires and assessments were included (see **Supplementary Tables 1 and 6** for the distribution of items across categories in the core data group and the final model, respectively). One questionnaire which was almost entirely excluded from modeling due to low N, Diet by 24-hour recall, contained 317 individual items relating to intake of certain foods over a 24-hour period. If this assessment had been included, we may have observed one or more factors more directly reflecting diet. This is also a hazard of trying to generalize our factor solution beyond the UK Biobank: beyond potential differences in sample makeup, the makeup of the items themselves would greatly influence resultant factor structure.

Impact of questionnaire structure

Surveys within the UK Biobank contained numerous questions for which a person may select either multiple responses (“categorical-multiple”) or only a single response (“categorical-single”). For example, a person may be asked to select as many vascular disorders as they have been previously diagnosed with, or to select only the type of milk that they most commonly consume. Typically these questions also include an option for “None of the above”. This is a common practice in surveys, and many in the lay public are familiar with such response patterns.

These types of questions pose an analytic problem for methods that rely on correlations across items, such as factor analysis, due to the constraints imposed by the survey question itself. For example, for both categorical-multiple and categorical-single question types, an answer of “None of the above” will necessarily be anticorrelated with dummy variables created for the other responses, resulting in a correlation structure that is entirely dependent upon what other options were included in the question. We observe this induced correlation structure in the results of our initial EFA, where items indicating a response of “None of the above” to a question were consistently among the top-loading items for factors. Factor 13, for example began with two “None of the above” responses to categorical-multiple questions involving use of supplements (e.g., “Vitamin and mineral supplements: None of the above” and “Mineral and other dietary supplements: None of the above”). The Factor consisted mostly of other responses from those two questions. When moving into an EFA framework, the “None of the above” items almost universally caused issues with model fit in the form of Heywood cases, and we therefore removed all of them (see **Differences between EFA and CFA** and **Supplementary Table 4**).

Categorical-single questions similarly force an anticorrelation across all possible responses. Returning to the example of milk type discussed above, a participant who most commonly drinks skimmed milk will necessarily not most commonly drink full cream or soy milk. Notably it's quite possible that for example favoring skimmed milk is similar to favoring semi-skimmed milk, in terms of underlying factors affecting milk preference or their correlation with would have traits (both diet and non-diet). Nevertheless the forced choice structure of the categorical-single question will prevent ever observing a positive correlation between skimmed and semi-skimmed milk preference.

Aside from questionnaire structure, this issue also arises from exclusionary diagnoses and medications. For example, individuals are commonly prescribed just one medication from a single class of medications. In these cases, though a group of individuals may have the same disease and associated conditions, each individual could be taking different medications. Therefore, though the disease and its associated conditions would independently be correlated with each of the medications, the medications themselves would be anticorrelated. Within our final CFA model, categorical-single items and medication codes within the same general group (e.g., the ACE inhibitors ramipril, perindopril, and lisinopril) consistently demonstrated the poorest pairwise residual misfit. Attempts to model the residual covariance between these items resulted in problems with the inversion of the information matrix (i.e., a nonpositive definite correlation matrix that then prevents inversion of information matrix) and would have presented an additional computational challenge in the calculation of latent factor scores for individuals. Alternatively, composite items could be created to combine these reported items that are

believed to be interchangeable into a single measure for e.g. taking an ACE inhibitor, but building such an item would instead require imposing assumptions on the relationship between the component items and how they each relate to other items in the factor analysis. Given the relatively low prevalence of such items in the overall factor model, we simply included these items “as is” in the analyses.

Extreme outliers on continuous variables

Though the primary Neale Lab UKB mega-GWAS results for continuous variables were reported for inverse-rank normal transformations, we used raw data values to facilitate easier comparison between the core and full EUR data groups. However, when moving from the EFA to the CFA framework, we noticed that the top modification indices for the model, which are meant to suggest alterations which would improve model fit, were dominated by continuous items with extreme outliers. This suggests that these extreme outliers (i.e., >20 standard deviations from the mean) were at least in part driving some of the correlation structure. To reduce this influence, we subsequently dropped from the core data group any individuals with a value >20 standard deviations from the mean on any continuous variable (N=65). Depending on whether these outliers reflect true values or data errors, this choice to stabilize the model risks reducing generalizability to individuals with truly extreme values. On the other hand it is unlikely that the phenotypic structure for such individuals would be well modeled regardless of their inclusion. We also note that these outliers still influence our current results, since excluding these outliers for CFA does not eliminate their impact on the structure selected from the EFA, and we chose not to rerun the EFA excluding these 65 individuals.

Modeling of non-continuous items

The items in the UK Biobank were derived from many different survey and assessment types, from self-report to verbal interview to medical diagnosis to biometric measurement. UKB therefore contained many different variable types, including continuous, binary, and ordinal, which is expected for any sort of large-scale deep-phenotyping biobank. Within the EFA, to arrive at the initial factor structure, we chose to treat all variables as continuous, consistent with the use of linear regression for GWAS in the Neale Lab UKB Round 2 mega-GWAS. However, treatment of ordered variables as continuous will necessarily misestimate their correlation. In the CFA, we therefore treated all variables as the appropriate data type: 88 as continuous, 346 as binary, and 130 as ordinal, and we considered the ordered variables as thresholds of a continuous liability distribution. Such a conversion seemed appropriate for use in these cases, as diagnoses are often conceptualized as artificial symptom thresholds imposed upon an underlying liability distribution.

Forced orthogonality between factors

For modeling purposes, with an eye towards computational scale and downstream analyses, we forced all factors to be orthogonal in both the EFA and CFA. Notably, though the *latent* factors were specified as orthogonal, the *observed* factor scores (generated in our case using extensions of the Bartlett and Thomson-Thurstone methods) were not necessarily orthogonal. Nonetheless, the highest pairwise correlation between factor scores was 0.176 (i.e., between F4 and F33; mean correlation=0.001[0.044]; see **Supplementary Figure 8**).

Orthogonality likely does not reflect the "true" behavior of latent constructs, and oblique rotations are generally favored with factor analysis. Our choice of such an orthogonal rotation therefore has some important implications for interpretation of our factors. Specifically, each factor must be viewed as representing the covariance structure of the items within it, *once accounting for covariance modeled by the other factors*.

A useful illustration can be found within the four factors most directly related to cardiometabolic disease, Factors 7, 12, 16, and 28. These factors can be broadly characterized as containing items related to BMI and adiposity (Factor 7), hypertension (Factor 12), coronary artery disease (Factor 16), and diabetes (Factor 28). In the "real world," the variance captured by each of these factors would likely be related; however, within our orthogonal model, we have explicitly required them to be unrelated. As such, Factor 28 could most accurately be interpreted as representing the remaining covariance of the items within it (e.g., mostly reflective of a diabetes diagnosis) once accounting for the variance explained by the other related (and unrelated) factors. The impacts of this orthogonalization can be demonstrated by comparing the GWAS of Factor 28 to a prior GWAS of type 2 diabetes (r_g=0.68[0.02]; [56]): the factor has higher genetic overlap with cholesterol measures (e.g., total cholesterol r_g=0.29 vs. 0.04; [57]) but lower overlap with BMI (r_g=0.23 vs. 0.49; [58]) and an inverse correlation with blood pressure (r_g=-0.18 vs. 0.20; [59]), reflective of its inclusion of high cholesterol in its factor definition and its independence of Factors 7 and 12 described above (**Extended Data Figure 8**). It is therefore critical to not assign meaning to the latent constructs identified by these analyses, or to their underlying genetic etiology, beyond seeing them as useful tools for interrogating potentially relevant axes of phenotypic variation across individuals.

Finally, as shown in **Supplementary Figure 8**, requiring the underlying latent phenotypic factors to be orthogonal (and, consequently, for the most part, for the factor scores to be uncorrelated) does not imply that they are genetically uncorrelated. For cases in which the factors are genetically correlated but phenotypically uncorrelated, they would have non-genetic (e.g., environmental) correlations counter-balancing their genetic correlation. However, we believe this conflicting pattern of association to be primarily driven by statistical artifacts of orthogonalization and thus caution against any biological interpretation.

Selection of "nuisance" covariates

Within both the EFA and CFA, we chose to residualize observed variables for all covariates used in the Neale Lab UKB Round 2 mega-GWAS: the first 20 genetic PCs, age, chromosomal sex, age^2, age-x-chromosomal sex, and age^2-x-chromosomal sex. We purposely selected these "nuisance" covariates to be consistent with the prior work of our collaborators, and also to avoid the identification of factors driven by these covariates. Put another way, we wanted to identify consistent axes of variation across the entire EUR subset of the UKB, regardless of chromosomal sex, age, or [EUR] ancestry. One could argue for the inclusion of additional "nuisance" regressors, such as assessment center or measures of socioeconomic status, depending on the intentions and goals of the analysis. In our own analysis, at least one factor, Factor 33, seems to recapitulate some aspects of regional clustering via assessment center. Items within that factor include current home and place-of-birth geographic coordinates, variables related to accommodation and heating types, and certain likely cultural food choices, such as preference for ground vs. instant coffee and weekly intake of both champagne and white wine. Scores on this factor

were generally associated with distance from London, and were highest among those at assessment centers in the immediate area.

Assumptions of factor invariance

Factor analysis relies on the assumption that the measurement model, or the relationship between the observed and latent variables, is equivalent across subgroups. These subgroups may be split by demographics (e.g., gender or assessment center) or, in the special case of our analyses, patterns of response. This assumption is unlikely to hold across all subgroups, and a number of methods exist to test for such differences[60]. The presence of a reasonably assessment-center-specific factor, described in the previous section, as well as differences in demographics between the core and full EUR groups (see **Characteristics of final core data group**) provide further evidence that this assumption is violated in practice. Additionally, our calculation of factor scores in the presence of missingness explicitly assumes that the covariance structure is the same across individuals regardless of which observed items were actually measured. Caution must thus be exercised in generalizing the model across subgroups. Conversely, a particularly fruitful avenue of future research may be to explicitly model and test for differences across subgroups.

Impact of structured missingness

A major goal of this project was to fairly comprehensively model the phenotypic landscape captured by UKB; as such, we sought to include as many assessments as possible to uncover relationships between variables not grouped a priori. However, this decision introduced the issue of structured missingness across assessments, as not all individuals

were given or responded to every assessment. To address this, we utilized a multi-stage approach in which the factor model was constructed based on a subsample of individuals with high assessment-level completeness, and then we scored the full EUR sample based on the parameters estimated within that subgroup. As mentioned in the prior section, this approach relies on the assumption of measurement invariance across the core and full subgroups.

Beyond special considerations given to the factor model in the context of structured missingness, problems arose when attempting to estimate factors scores in individuals for which the majority of variance in the latent score was missing. Though our factor scoring method is able to estimate scores in the context of item-level missingness, relying on expected patterns of covariance between the missing and nonmissing items, it cannot "recover" variance when the majority of items within a factor are missing, as in the case of assessment-level missingness. For example, Factor 24 is based primarily on items from an empirical eye assessment which was introduced later in the UKB battery (i.e., 14 of 17 factor items are from that one assessment). As such, individuals who are missing that assessment do not have enough measured indicators to reasonably estimate a value for that latent factor. To account for this, calculated for each missingness pattern the amount of variance explained by the items available versus a hypothetical Bartlett factor score with no missingness. For inclusion in further analyses, we required that individuals have nonmisssing items with the ability to explain at least 80% of variance in that hypothetical factor score. This thresholding severely reduced our sample sizes for a number of factors (e.g., N=75,226 for Factor 24; **Supplementary Figure 6**) but was necessary to ensure some reasonable degree of comparison across individuals with

different missingness patterns. Even with this restriction our factor score regression results may be biased, especially for analyses with the independent variable factor scores, depending on whether the estimation error in the factor scores as a function of the different missingness patterns is correlated with the other terms in the regression models.

Structured missingness thus has the ability to impact both the estimation of the factor model and individual-level latent factor scores, and extensive consideration must be given to how to reduce its impact if it is necessary to the research question at hand.

Latent causal variable analysis

To evaluate whether we can distinguish which items in a factor are more likely to be causes or consequences of the identified structure we performed latent causal variable (LCV; [61]) analysis of their genetic results. Briefly, using genome-wide results for a given pair of genetically correlated traits LCV tests whether there's evidence for a causal relationship between the pair based on higher-order moments of their GWAS Z scores. Each relationship is characterized by an estimate of the genetic causality proportion (gcp), with values ranging from 1 (meaning the genetics of the first trait have a causal effect on the second trait), to -1 (meaning conversely that the second trait's genetics causes the first trait), with gcp=0 indicating no casual relationship. Using this approach, we evaluate the relationship between the top items in factor for four selected factors of interest (6, 11, 16, and 23).

Results of the LCV analyses are shown in **Supplementary Figure 10**. Broadly, we observe that there is limited power to distinguish causal directions between the top items

based on their UK Biobank GWAS results (i.e., the standard errors are generally large). Nevertheless, we do find evidence of partial genetic causality in two instances: an effect of diagnosed asthma and other top items in Factor 11 on reported breathing problems during a job (asthma gcp=0.80, p=3.98e-5), and an effect of ICD diagnosis of chronic ischemic heart disease on ICD diagnosis of myocardial infarction. Nominal evidence is also found for a couple other relationships, including a partially causal effect of participation in strenuous sports on overall health rating (gcp=0.37, p=5.0e-3), but they do not survive correction for multiple testing.

Overall, these results suggest that complete causality (gcp=1 or -1) between the items in the current study are unlikely, but that there will likely be the potential to identify partially causal relationships at larger GWAS sample sizes. We also observed that the relationships likely to be only be partially causal, consistent with the existence of some shared genetic components across items along with other genetic risk factors that are more item-specific.

Tissue specificity of shared and nonshared genetic effects

Based on the observation that some genetic loci are significantly associated with some of the top items in a factor without being associated with the factor itself (**Figure 3C**), we consider whether the more item-specific genetic effects correspond to different tissues or cell types than factor-associated effects. Such a difference would suggest that the observed item-specific loci reflect different genetic mechanisms or processes that aren't fully shared across the factor. We assess this possibility for each of the top 5 items in

Factors 11 and 16, respectively, since both have known relevant tissues and multiple loci significantly associated with the top items that aren't observed in the corresponding factor GWAS.

Methods

To evaluate this possibility, we first use Genomic Structural Equation Modeling (gSEM[62]) to estimate genetic effects for a given item controlling for the shared genetic effect present in the GWAS of the factor. The item-specific effects are estimated for each SNP based on a GWAS-by-subtraction model[63]. The structural part of the model is estimated using the GWAS results for HapMap3 SNPs along with reference panel LD scores previously computed from European ancestry individuals from the 1000 Genomes Project[64]. We compare genome-wide significant loci from this GWAS-by-subtraction for a given item to the GWAS of the factor to identify potentially item-specific locus associations.

The GWAS-by-subtraction results for each item are then tested for tissue and cell type enrichments using LD score regression[65]. We consider a total of 694 annotations based on gene expression and chromatin marks from ROADMAP, ENCODE, GTEx, and Franke lab, each tested conditional on the baseline LD v1.2 model controlling for annotated functional categories, LD-related genomic features, and MAF[66]. For each item, the LD score regression for each cell type is compared to the corresponding cell type coefficient from LD score regression of the factor GWAS after standardizing the coefficients based on the estimated total observed scale SNP-heritability for the GWAS-by-subtraction of the item and the GWAS of the factor, respectively. Pairwise comparison between the

estimated cell type coefficients $\hat{\tau}$ from the item and the factor results is done for each tissue/cell type using the two-sided z-score

$$z_{cell} = \frac{\hat{\tau}_{cell,factor} - \hat{\tau}_{cell,item}}{\sqrt{SE(\hat{\tau}_{cell,factor})^2 + SE(\hat{\tau}_{cell,item})^2}}$$

The general trend across all 694 annotations is then tested using Deming regression to account for the estimated standard errors on each estimate.

Results

After GWAS-by-subtraction with genomic SEM, 3 of the top 5 items from Factor 11 and none of the top 5 items from Factor 16 had genome-wide significant loci ($p < 5e-8$) remaining. The observed loci from Factor 11 items, however, were all loci previously observed to be strongly associated with both the item and the factor, with consistently weaker results in the conditional analysis. This could indicate either incomplete control for shared effects in the GWAS-by-subtraction model or differential effects at shared loci. In either case, these results do not identify any distinctly item-specific loci reaching significance in the current results.

Across the 10 tested items, a total of 19 tissue-item pairs have significantly enriched genetic signal after Bonferroni correction within each item ($p < 7.2e-5 = .05/694$). A majority of the significant enrichments (11) are from regions with chromatin marks associated with T cells in the GWAS-by-subtraction of self-report of asthma or hayfever/allergic rhinitis/eczema diagnoses (UKB code 6152) in Factor 11. On the other hand, 18 of the 19 tissues, including all of the T cell annotations, also have significant

enrichments in LD score regression of the corresponding factor GWAS. The one exception is for an enrichment related to regions of gene expression in blood vessels (A07.231.Blood.Vessels) which is significantly enriched after GWAS-by-subtraction of myocardial infarction (ICD code I21; p=6.3e-5) but only nominally enriched in the GWAS of factor 16 (p=.039). The difference in the coefficient between these results is not significant though (standardized factor coefficient = 7.10e-8 [SE=4.04e-8], item coefficient = 1.99e-7 [SE=5.20e-8], difference p=.051).

More broadly, the tissue/cell-type coefficients from the GWAS-by-subtraction results for item-specific genetic signal are highly concordant with the tissue/cell-type results for the GWAS of the corresponding factors (e.g. **Supplementary Figure 9A**). Across all items, no tissues/cell types have significantly different enrichment coefficients between the item-specific and factor GWASs after correction for multiple testing. Furthermore, for 9 of the 10 items the observed results are consistent with perfect correlation in cell type results between the factor and GWAS-by-subtraction of the item (p > 0.5 for Deming regression model goodness of fit). The exception is breathing problems during a job period (UKB code 22616), whose item-specific GWAS shows much less consistency in tissue/cell-type enrichments compared to Factor 11 (p=1.45e-4; **Supplementary Figure 9B**). Still, no individual cell type reaches Bonferroni-corrected significance for this comparison.

Taken together, these results do not identify any clear item-specific loci or patterns of enrichment among the top items in Factors 11 and 16. To the extent that there is any genetic signal remaining after controlling for the shared signal through GWAS-by-subtraction there is some evidence of loci and tissues that are shared with the factor but may have differential effects on some items. On the other hand it is difficult to distinguish

true differential effects of this sort from incomplete control of the shared effects in the gSEM modeling, so the remaining effects should be interpreted with caution. We also note that given the items are highly correlated with the factors (by design) there is generally low remaining SNP-heritability in the GWAS-by-subtraction results after controlling for the factor (observed scale h^2_g=0.0011-0.0693 in top 5 Factor 11 items, h^2_g=0.00002-0.0036 in Factor 16 items), limiting power to identify any enrichments. Thus it remains possible that item-specific loci and mechanisms exist, both for these items or in other factors we have not evaluated here, but that we lack sufficient power to identify them in the current data.

Genetic correlations between prior SES GWAS and all factors

Broader comparisons with the prior SES GWAS indicate that our reduced-rank representation of the phenome captured by UKB does not fully capture genetic associations with SES in only these 3 factors (i.e., Factors 5, 10, and 15; **Supplementary Figure 11**). We observe, for example, strong genetic correlations of Factor 4 and Factor 33, containing items related to urbanicity and living near London, respectively, with GWAS of regional social deprivation[67] (Factor 4 r_g=0.93[0.08]; Factor 33 r_g=-0.47[0.09]). The results also reflect how our model of uncorrelated factors attempts to partition signal for different aspects of conventional SES measures; for example, non-parental effects on educational attainment[68] are genetically correlated not only with Factors 5 and 10, but also with Factor 34, which contains items related to cognition and processing speed (r_g=0.60[0.17]). Residual genetic correlation is also observed between parental effects on educational attainment and Factors 20 (e.g., severe, life-threatening illness, r_g=-

0.45[0.10]), 31 (e.g., gastrointestinal issues, r_g=-0.55[0.11]), and 32 (e.g., hearing difficulties, r_g=-0.47[0.14]), and between social deprivation and Factors 6 (e.g., smoking and associated risk behaviors, r_g=0.52[0.05]) and 9 (e.g., trauma, r_g=0.55[0.06]), further reflecting the complex interconnections between varied SES measures and health.

References

1. Bycroft, C. *et al.* The UK Biobank resource with deep phenotyping and genomic data. *Nature* **562**, 203–209 (2018).

2. Adams, M. J. *et al.* Factors associated with sharing e-mail information and mental health survey participation in large population cohorts. *Int. J. Epidemiol.* **49**, 410–421 (2020).

3. Omura, G. Correlates of item nonresponse. *J. Mark. Res. Soc.* (1983).

4. Mignogna, G. *et al.* Patterns of item nonresponse behaviour to survey questionnaires are systematic and associated with genetic loci. *Nat. Hum. Behav. 2023* 1–17 (2023) doi:10.1038/s41562-023-01632-7.

5. Revelle, W. psych: Procedures for personality and psychological research. (2015).

6. Brown, J. Choosing the right number of components or factors in PCA and EFA. *JALT Test. Eval. SIG Newsl.* **13**, (2009).

7. Zwick, W. R. & Velicer, W. F. Comparison of Five Rules for Determining the Number of Components to Retain. *Psychol. Bull.* **99**, 432–442 (1986).

8. Kaiser, H. F. The Application of Electronic Computers to Factor Analysis. *Educ. Psychol. Meas.* **20**, 141–151 (1960).

9. Horn, J. L. A rationale and test for the number of factors in factor analysis. *Psychometrika* **30**, 179–185 (1965).

10. Cattell, R. B. *The scientific use of factor analysis in behavioral and life sciences*. (Springer US, 1978). doi:10.1007/978-1-4684-2262-7.

11. Velicer, W. F. & Jackson, D. N. Component Analysis versus Common Factor Analysis: Some issues in Selecting an Appropriate Procedure. *Multivariate Behav. Res.* **25**, 1–28 (1990).

12. Thurstone, L. L. The vectors of mind. *Psychol. Rev.* **41**, 1–32 (1934).

13. Jolliffe, I. T. *Principal Component Analysis*. (Springer, 2002).

14. Harman, H. H. & Jones, W. H. Factor analysis by minimizing residuals (minres). *Psychometrika* **31**, 351–368 (1966).

15. Patterson, N., Price, A. L. & Reich, D. Population structure and eigenanalysis. *PLoS Genet.* **2**, 2074–2093 (2006).

16. Zhang, Z., Chan, K. L., Kwok, J. T. & Yeung, D.-Y. Bayesian Inference on Principal Component Analysis using Reversible Jump Markov Chain Monte Carlo. *Proc. Ninet. Natl. Conf. Artif. Intell. Sixt. Innov. Appl. Artif. Intell. Conf.* 372 (2004).

17. Kaiser, H. F. The varimax criterion for analytic rotation in factor analysis. *Psychometrika* **23**, 187–200 (1958).

18. Rosseel, Y. lavaan: An R Package for Structural Equation Modeling. *J. Stat. Softw.* **48**, 1–36 (2012).

19. Van Buuren, S. & Groothuis-Oudshoorn, K. mice: Multivariate imputation by chained equations in R. *J. Stat. Softw.* **45**, 1–67 (2011).

20. DiStefano, C., McDaniel, H. L., Zhang, L., Shi, D. & Jiang, Z. Fitting Large Factor Analysis Models With OrdinalData. *Educ. Psychol. Meas.* **79**, 417 (2019).

21. Jöreskog, K. G. & Moustaki, I. Factor Analysis of Ordinal Variables: A Comparison of Three Approaches. *Multivariate Behav. Res.* **36**, 347–387 (2001).

22. Brown, T. *Confirmatory factor analysis for applied research*. (The Guilford Press, 2015).

23. Kyriazos, T., Poga-Kyriazou, M., Kyriazos, T. & Poga-Kyriazou, M. Applied Psychometrics: Estimator Considerations in Commonly Encountered Conditions in CFA, SEM, and EFA Practice. *Psychology* **14**, 799–828 (2023).

24. Wang, J. & Wang, X. *Structural Equation Modeling : Applications Using Mplus.* (John Wiley & Sons, Incorporated, 2019).

25. Muthén, B. A general structural equation model with dichotomous, ordered categorical, and continuous latent variable indicators. *Psychometrika* **49**, 115–132 (1984).

26. Bates, D. & Maechler, M. Matrix: Sparse and dense matrix classes and methods. (2010).

27. Privé, F., Aschard, H., Ziyatdinov, A. & Blum, M. G. Efficient analysis of large-scale genome-wide data with two R packages: bigstatsr and bigsnpr. *Bioinformatics* **34**, 2781–2787 (2018).

28. Daniel, F., Weston, S. & Tenenbaum, D. doParallel: Foreach Parallel Adaptor for the ‘parallel’ Package. (2015).

29. Wirth, R. J. & Edwards, M. C. Item Factor Analysis: Current Approaches and Future Directions. *Psychol. Methods* **12**, 58 (2007).

30. Hunter, M. D., Pritikin, J. N., Kirkpatrick, R. M. & Neale, M. C. Rethinking Ordinal Variable Identification in Weighted Least Squares Structural Equation Modeling. doi:10.31234/OSF.IO/MNC7Q.

31. Lai, K. & Green, S. B. The Problem with Having Two Watches: Assessment of Fit When RMSEA and CFI Disagree. *Multivariate Behav. Res.* **51**, 220–239 (2016).

32. Savalei, V. Improving Fit Indices in Structural Equation Modeling with Categorical Data. *Multivariate Behav. Res.* **56**, 390–407 (2020).

33. Shi, D., Lee, T. & Maydeu-Olivares, A. Understanding the Model Size Effect on SEM Fit Indices. *Educ. Psychol. Meas.* **79**, 310 (2019).

34. Bartlett, M. S. Smoothing Periodograms from Time-Series with Continuous Spectra. *Nature* **161**, 686–687 (1948).

35. Bartlett, M. S. Periodogram analysis and continuous spectra. *Biometrika* **37**, 1–16 (1950).

36. Thomson, G. H. The meaning of ‘i’ in the estimate of ‘g’. *Br. J. Psychol. Gen. Sect.* **25**, 92–99 (1934).

37. Skrondal, A. & Laake, P. Regression among factor scores. *Psychom. 2001 664* **66**, 563–575 (2001).

38. Devlieger, I., Mayer, A. & Rosseel, Y. Hypothesis Testing Using Factor Score Regression: A Comparison of Four Methods. *Educ. Psychol. Meas.* **76**, 741 (2016).

39. Bartlett, M. S. The statistical conception of mental factors. *Br. J. Psychol. Gen. Sect.* **28**, 97–104 (1937).

40. Henderson, H. V. & Searle, S. R. On Deriving the Inverse of a Sum of Matrices. *SIAM Rev.* **23**, 53–60 (2012).

41. Lawley, D. N. & Maxwell, A. E. Factor Analysis as a Statistical Method. *Stat.* **12**, 209 (1962).

42. Duncan, W. J. LXXVIII. Some devices for the solution of large sets of simultaneous linear equations: With an appendix on the reciprocation of partitioned matrices. *London, Edinburgh, Dublin Philos. Mag. J. Sci.* **35**, 660–670 (1944).

43. Krijnen, W. P., Wansbeek, T. & Ten Berge, J. M. F. Best linear predictors for factor scores. *Commun. Stat. - Theory Methods* **25**, 3013–3025 (1996).

44. Cox, N. R. Estimation of the Correlation between a Continuous and a Discrete Variable. *Biometrics* **30**, 171 (1974).

45. White, H. A Heteroskedasticity-Consistent Covariance Matrix Estimator and a Direct Test for Heteroskedasticity. *Econometrica* **48**, 817 (1980).

46. MacKinnon, J. G. & White, H. Some heteroskedasticity-consistent covariance matrix estimators with improved finite sample properties. *J. Econom.* **29**, 305–325 (1985).

47. Long, J. S. & Ervin, L. H. Using Heteroscedasticity Consistent Standard Errors in the Linear Regression Model. *Am. Stat.* **54**, 217 (2000).

48. Huang, Y. & Wang, C. Y. Cox Regression with Dependent Error in Covariates. *Biometrics* **74**, 118 (2018).

49. Li, Y. & Ryan, L. Survival Analysis With Heterogeneous Covariate Measurement Error. *J. Am. Stat. Assoc.* **99**, 724–735 (2004).

50. Augustin, T. An Exact Corrected Log-Likelihood Function for Cox's Proportional Hazards Model under Measurement Error and Some Extensions. *Scand. J. Stat.* **31**, 43–50 (2004).

51. Golub, G. H. & Loan, C. F. van. An Analysis of the Total Least Squares Problem. *SIAM J. Numer. Anal.* **17**, 883–893 (1980).

52. Bekker, P. A. Alternative Approximations to the Distributions of Instrumental Variable Estimators. *Econometrica* **62**, 657 (1994).

53. Yuan, K.-H., Bentler, P. M. & Kano, Y. On Averaging Variables in a Confirmatory Factor Analysis Model. *Behaviormetrika* **24**, 71–83 (1997).

54. Bollen, K. A. *Structural equations with latent variables*. (John Wiley & Sons, 1989). doi:10.1002/9781118619179.

55. Harman, H. *Modern factor analysis*. (University of Chicago Press, 1976).

56. Mahajan, A. *et al.* Fine-mapping type 2 diabetes loci to single-variant resolution using high-density imputation and islet-specific epigenome maps. *Nat. Genet. 2018 5011* **50**, 1505–1513 (2018).

57. Willer, C. J. *et al.* Discovery and Refinement of Loci Associated with Lipid Levels. *Nat. Genet.* **45**, 1274 (2013).

58. Locke, A. E. *et al.* Genetic studies of body mass index yield new insights for

obesity biology. *Nature* **518**, 197 (2015).

59. Evangelou, E. *et al.* Genetic analysis of over 1 million people identifies 535 new loci associated with blood pressure traits. *Nat. Genet.* **50**, 1412–1425 (2018).

60. Millsap, R. E. Statistical approaches to measurement invariance. *Stat. Approaches to Meas. Invariance* 1–355 (2012) doi:10.4324/9780203821961.

61. O'Connor, L. J. & Price, A. L. Distinguishing genetic correlation from causation across 52 diseases and complex traits. *Nat. Genet. 2018 5012* **50**, 1728–1734 (2018).

62. Grotzinger, A. D. *et al.* Genomic structural equation modelling provides insights into the multivariate genetic architecture of complex traits. *Nat. Hum. Behav.* (2019) doi:10.1038/s41562-019-0566-x.

63. Demange, P. A. *et al.* Investigating the genetic architecture of non-cognitive skills using GWAS-by-subtraction. *Nat. Genet.* **53**, 35 (2021).

64. Bulik-Sullivan, B. *et al.* LD score regression distinguishes confounding from polygenicity in genome-wide association studies. *Nat. Genet.* **47**, 291–295 (2015).

65. Finucane, H. K. *et al.* Heritability enrichment of specifically expressed genes identifies disease-relevant tissues and cell types. *Nat. Genet.* **50**, (2018).

66. Gazal, S. *et al.* Linkage disequilibrium-dependent architecture of human complex traits shows action of negative selection. *Nat. Genet.* **49**, 1421–1427 (2017).

67. Hill, W. D. *et al.* Molecular Genetic Contributions to Social Deprivation and Household Income in UK Biobank. *Curr. Biol.* **26**, 3083 (2016).

68. Young, A. I. *et al.* Mendelian imputation of parental genotypes improves estimates of direct genetic effects. *Nat. Genet. 2022 546* **54**, 897–905 (2022).

Supplementary Figures

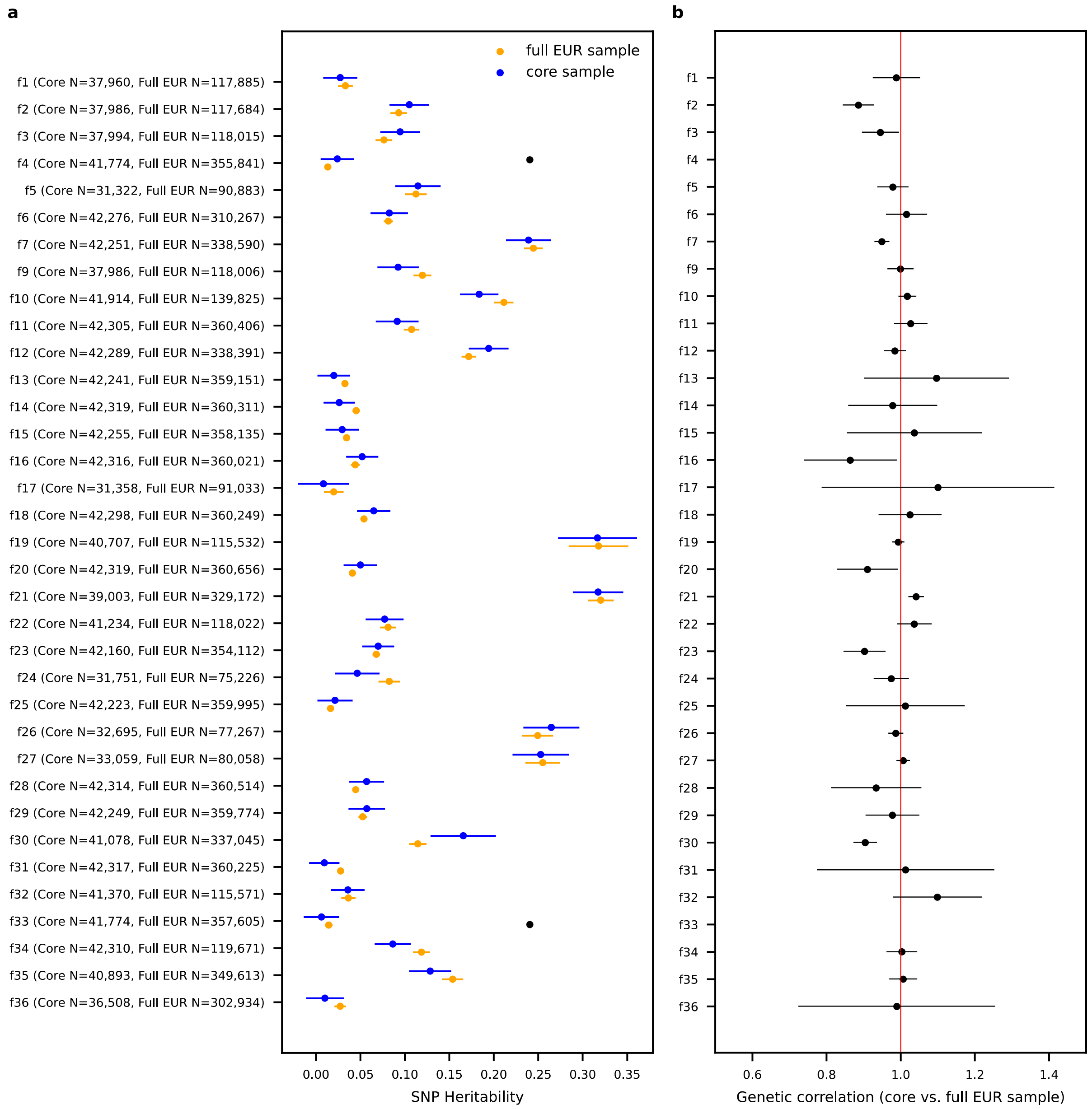

Supplementary Figure 1. Differences in genetic architecture between core and full-European-ancestry samples. Panel **A** shows the estimated SNP-heritability +/- 1 standard error for each factor in the core sample (in blue) versus the full EUR-ancestry sample (in orange). Panel **B** shows the point estimate of the genetic correlation +/- 1 standard error between the two samples. Genetic correlations that could not be estimated (e.g., due to lack of heritability in one of the samples) are shown as r_g=0 with no standard error estimate.

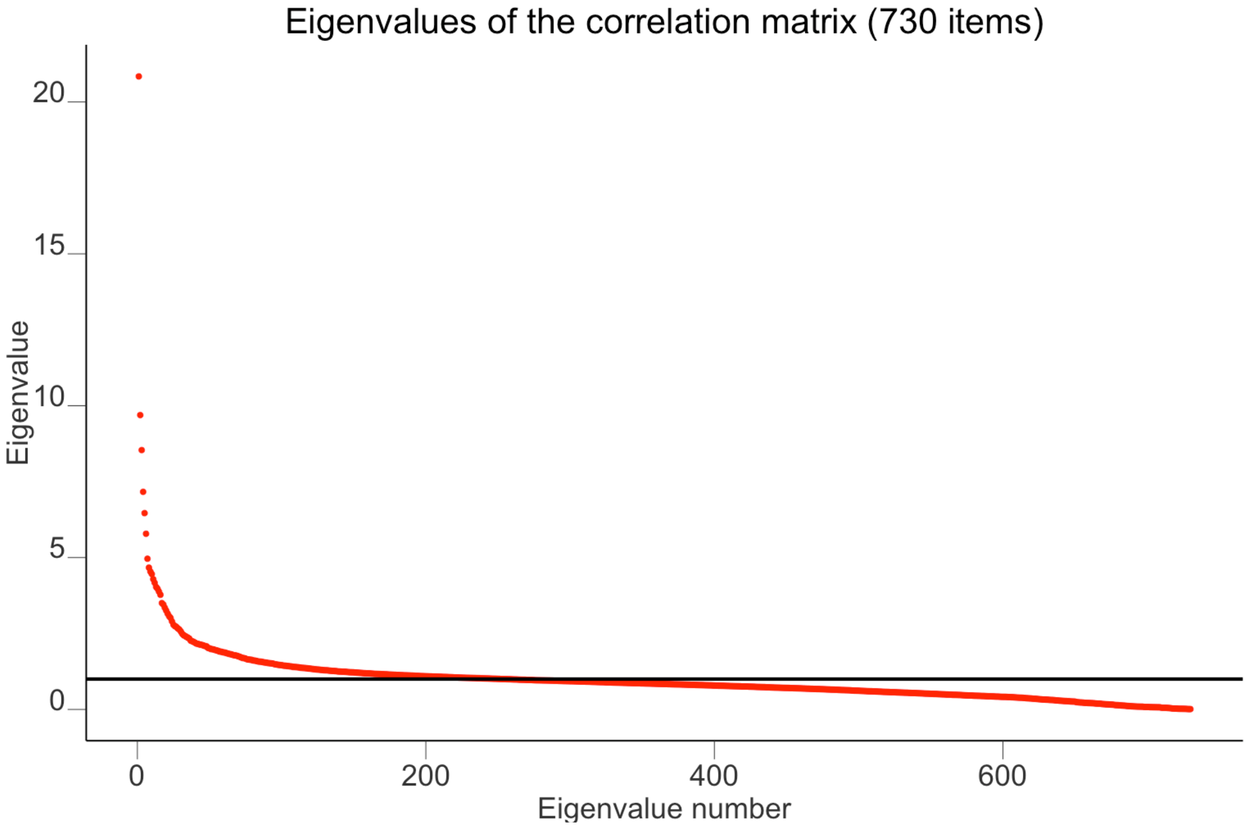

Supplementary Figure 2. Scree plot of eigenvalues of the correlation matrix used for exploratory factor analysis. The red dots show the 730 eigenvalues, and the horizontal dashed line corresponds to a value of 1.

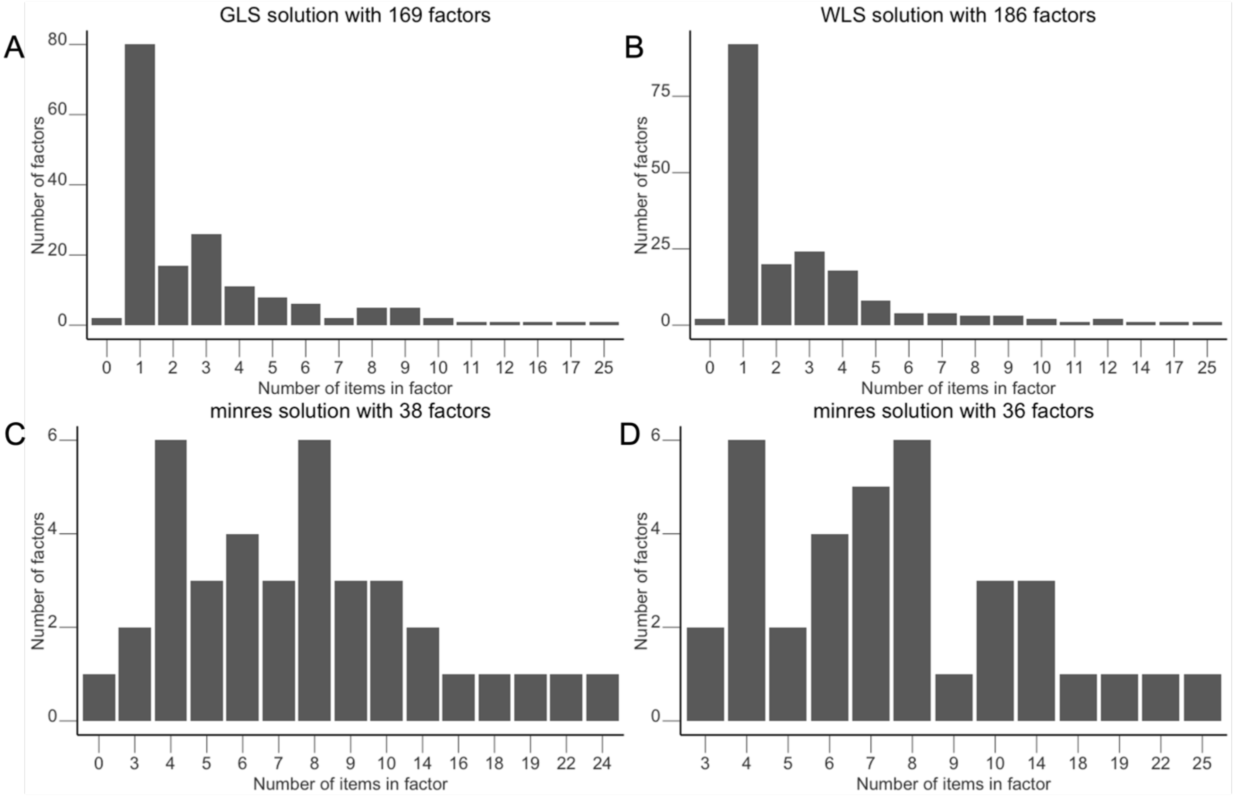

Supplementary Figure 3. Distribution of number of items in each factor for different factor models. A-C: GLS, WLS and MINRES methods with maximum number of factors (no Heywood or ultra-Heywood cases). **D:** final EFA solution of 36 factors using the MINRES method.

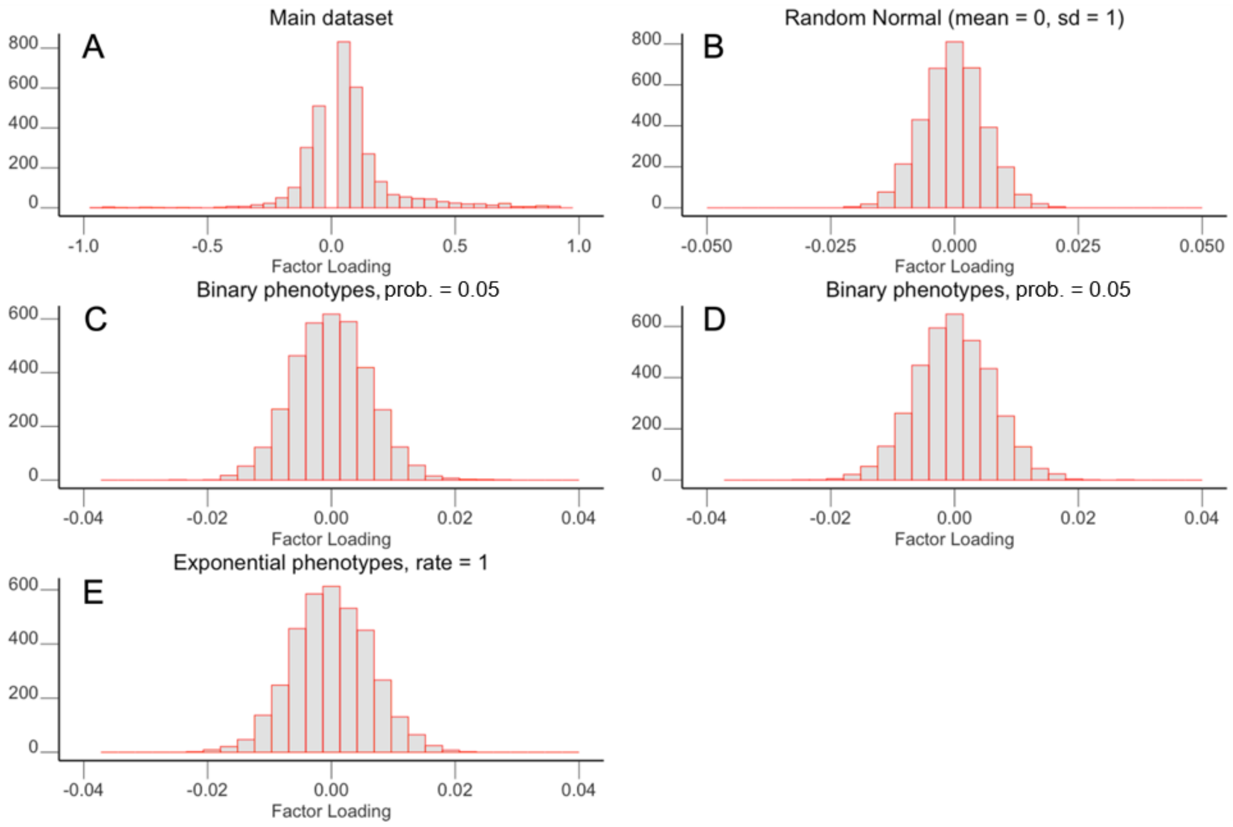

Supplementary Figure 4. Comparison of factor loading distributions of the MINRES-36 model for randomly generated items and real data. A) main dataset (730 items). Loadings between -0.05 and 0.05 were excluded to show the full range more clearly. **B)** Loadings for 100 random normally distributed traits. **C)** and **D)** Loadings for 100 binary traits (binomial distribution with probability = 0.05 and probability = 0.5, respectively). **E)** Loadings for 100 random exponential traits (rate = 1).

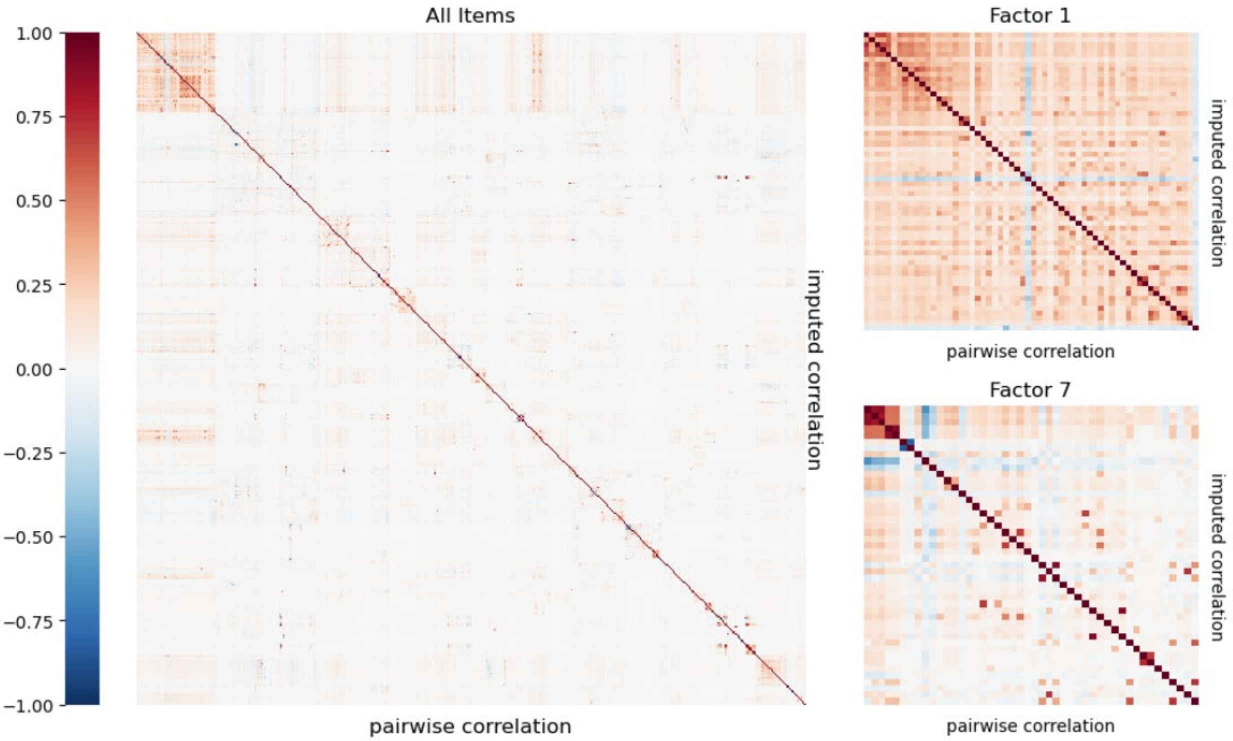

Supplementary Figure 5. Comparison of correlation matrices generated using pairwise-complete versus imputed data in the core sample. The lower triangle correlations were generated using pairwise deletion for missingness, while the upper triangle correlations were generated using complete data for a single imputation. Comparisons are shown across all items on the left, as well as within two representative factors (Factor 1 and Factor 7) on the right. Correlation magnitude and direction is indicated by color.

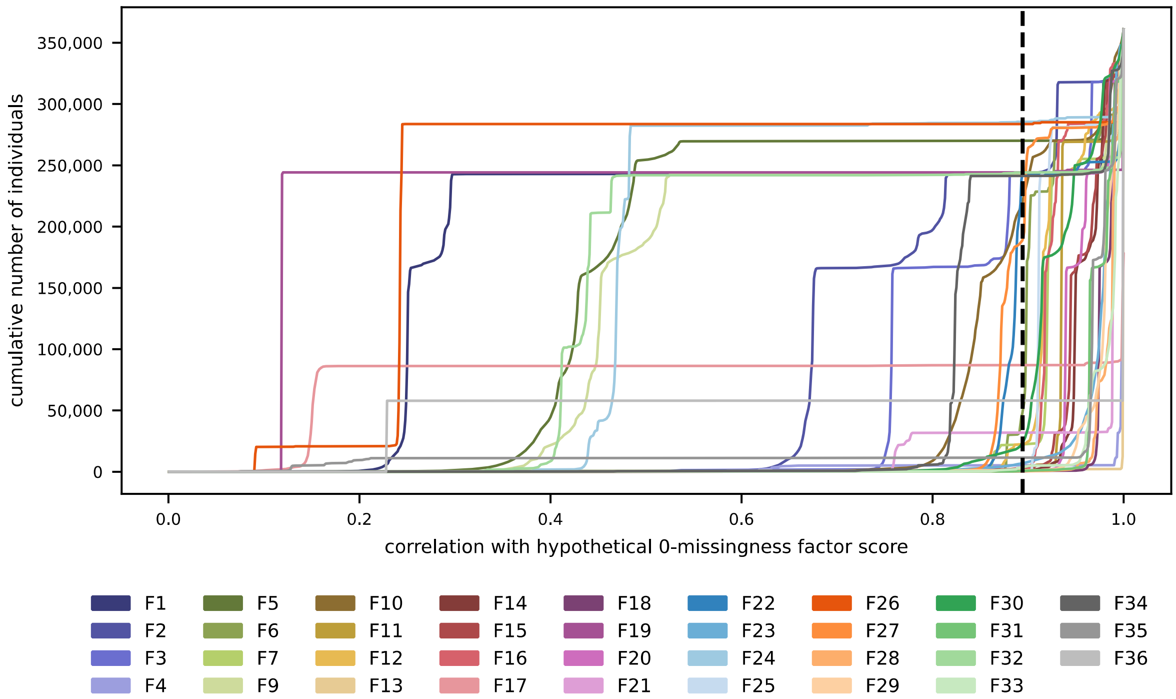

Supplementary Figure 6. Factor score thresholding to account for structured missingness. X-axis shows the correlation, for a given missingness pattern in the data, with a hypothetical 0-missingness Bartlett score. The y-axis shows the cumulative number of individuals per factor with a correlation value above that threshold. Major "jumps" in the data indicate the influence of structured missingness. The dashed black line represents our chosen threshold for inclusion, with an r^2>0.80.

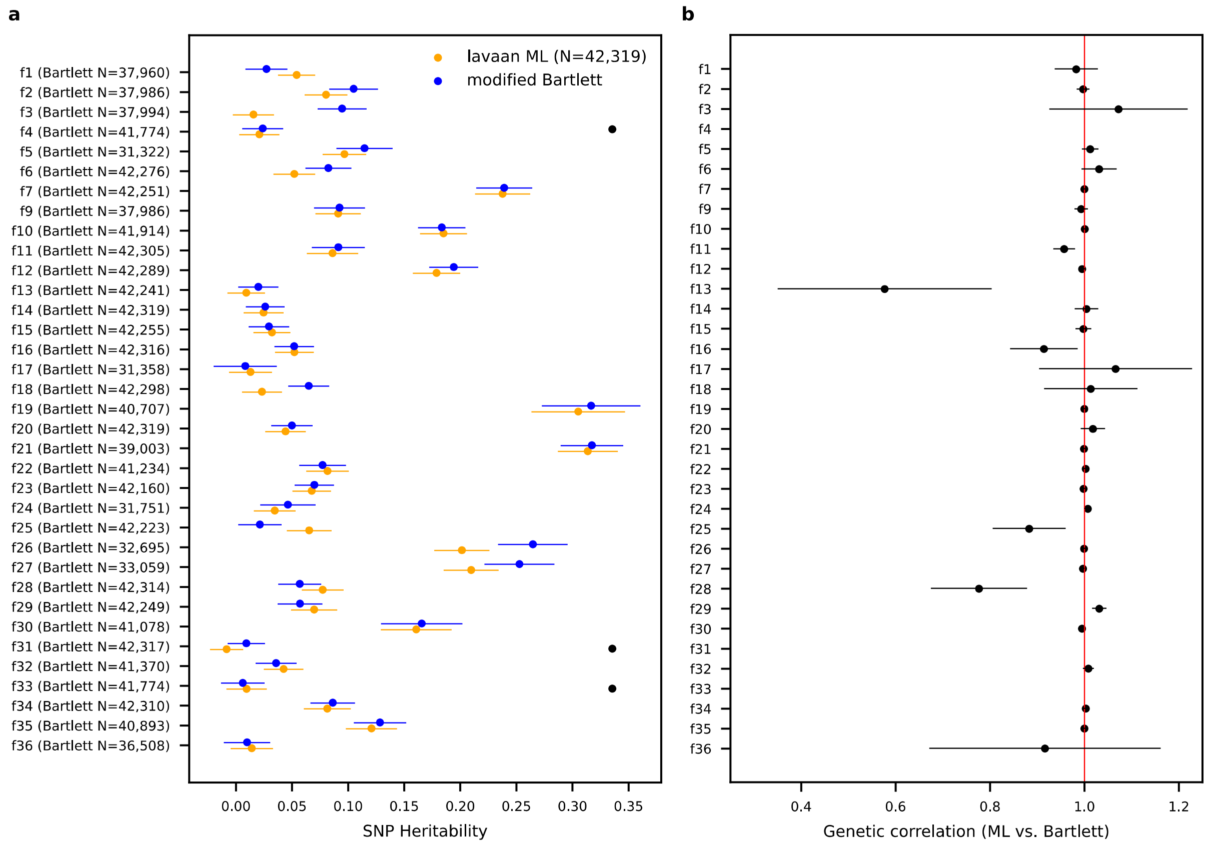

Supplementary Figure 7. Differences in genetic architecture between factor scores estimated using lavPredict and our adapted Bartlett scoring methodology. Panel **A** shows the estimated SNP-heritability +/- 1 standard error for each factor when factor scores were generated using our adapted Bartlett scoring methodology (in blue) versus using the default maximum likelihood (ML) estimator in lavaan (in orange), both in the core sample. Panel **B** shows the point estimate of the genetic correlation +/- 1 standard error between factor scores from the two methods in the core sample. Genetic correlations that could not be estimated (e.g., due to lack of heritability for at least one of the methods) are indicated by a dashed gray line.

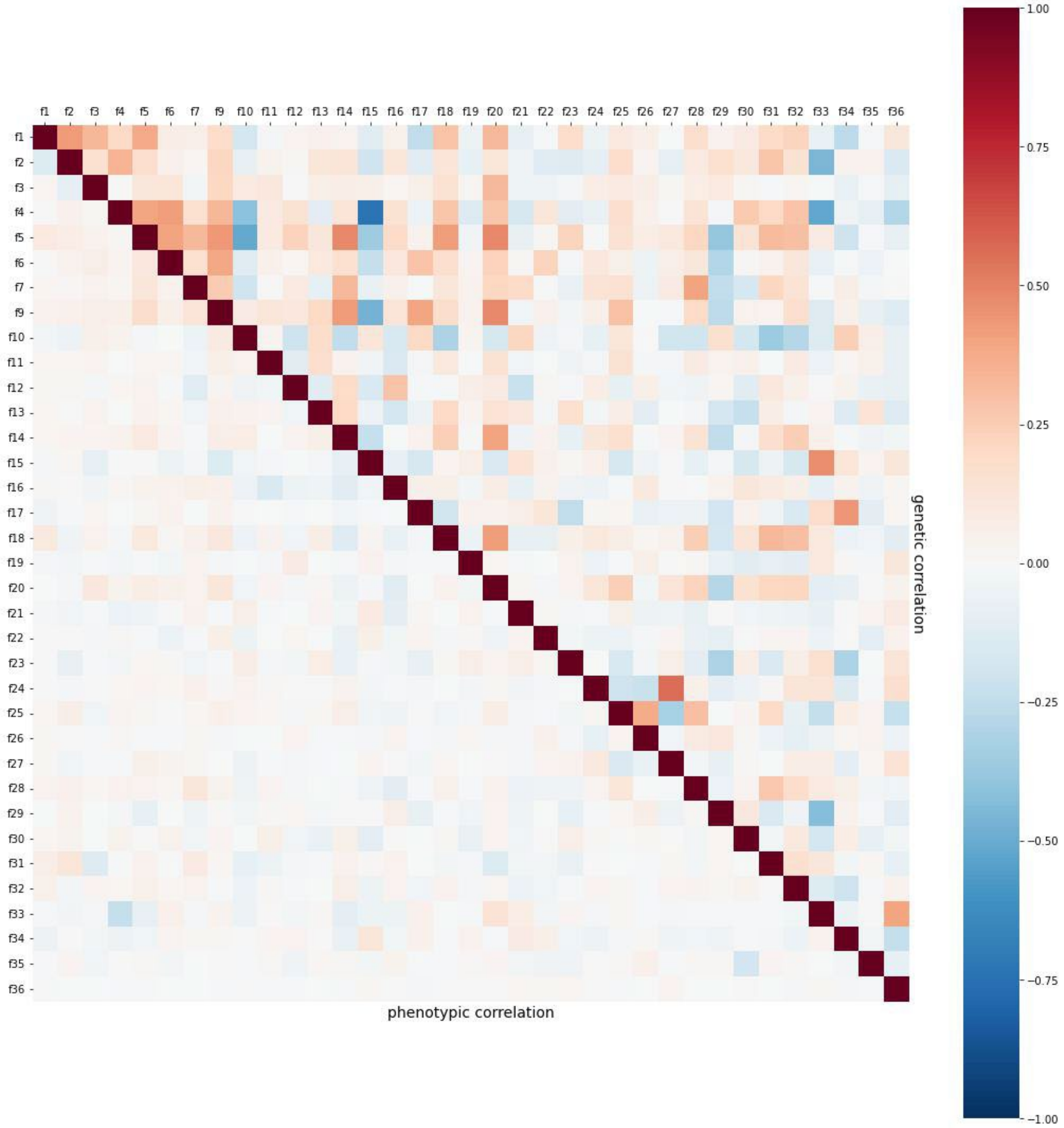

Supplementary Figure 8. Phenotypic and genetic correlations across factors. Phenotypic correlations between factors are shown in the lower triangle, and genetic correlations are shown in the upper triangle. Color indicates the magnitude and direction of correlation.

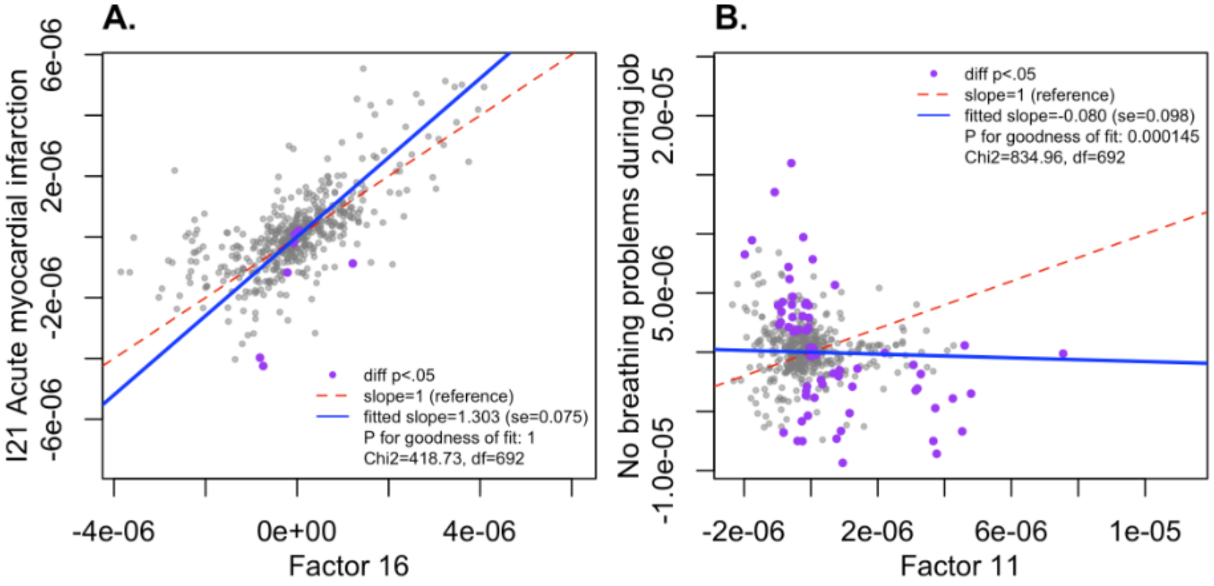

Supplementary Figure 9. Comparison of coefficients for cell/tissue type annotations between GWAS of factor scores and GWAS-by-subtraction of top items controlling for the corresponding factor. Coefficients are from LD score regression of 694 expression and chromatin-based annotations, scaled based on the total observed SNP heritability. The dashed red reference line indicates equal coefficients in the two analyses (slope = 1). Purple dots are nominally (unadjusted two-sided p<.05) different from the equality line based on a z-score test of the difference in the coefficients (**Supplementary Text**), but are not significant after multiple testing correction. The solid blue line indicates the results of Deming regression on the plotted data, accounting for the reported standard errors on the coefficients. Goodness of fit statistics for the Deming regression correspond to the null hypothesis that the true coefficients are perfectly correlated with measurement error equal to the reported standard errors.

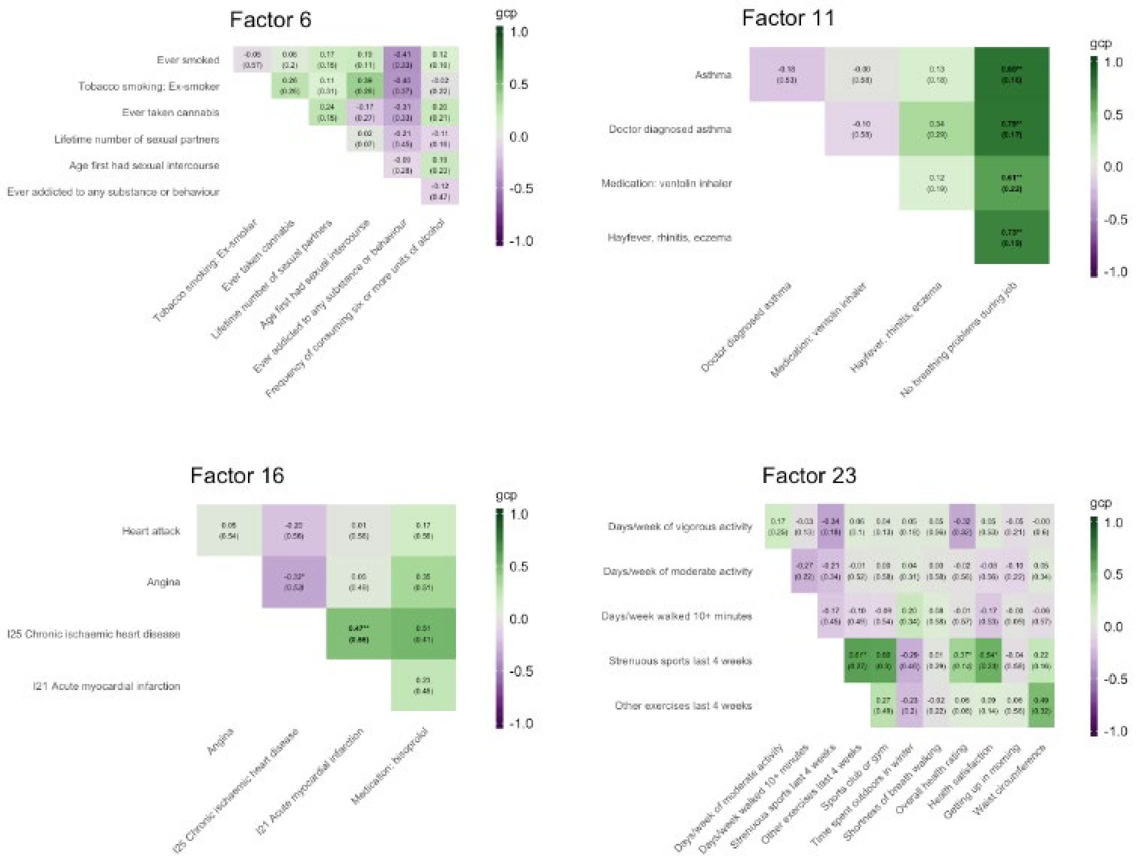

Supplementary Figure 10. LCV results for top items in factors 6, 11, 16, and 23. Genetic causal proportion (gcp) values are reported for each pair of items, along with their standard error. Values in italics with a single asterisk are nominally significantly different from 0 (two-sided unadjusted p<.05) based on the t-test of the LCV test statistic *S(0)*, and values in bold with two asterisks are significant after Bonferroni correction for the number of the trait pairs tested. A GCP value of 1 indicates that the genetic component of the item in the row is causal for the genetics of the item in the column. A GCP value of -1 indicates the reverse, that the item in the column is genetically causal for the item in the row. A GCP of 0 indicates that the pair of items share a latent cause. Intermediate values suggest "partial" causality, i.e. that some (but not all) elements of one item's genetics are causal for the other item.

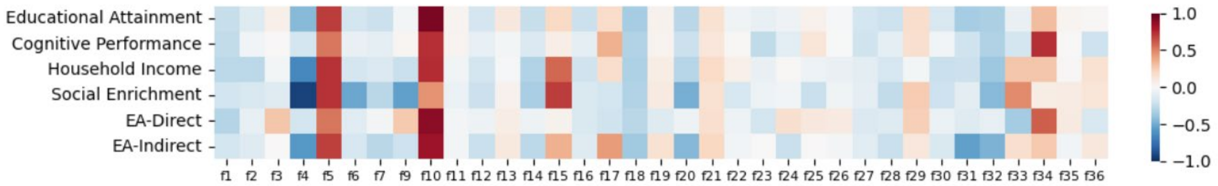

Supplementary Figure 11. Genetic overlap between prior GWAS of SES indicators and all factors. All genetic associations are flipped to be in the direction reflecting greater SES for consistency (e.g., “Social Deprivation” becomes “Social Enrichment”). Color of each box within the heatmap indicates the strength and directionality of genetic overlap across the two corresponding phenotypes. EA: Educational Attainment.
