# Supplementary material for: Principled distillation of UK Biobank phenotype data reveals underlying structure in human variation
Source: Nat Hum Behav. 2024 Jul 4;8(8):1599–615. doi: 10.1038/s41562-024-01909-5 (PMC11343713; doi:10.1038/s41562-024-01909-5)
Supplement: Supplementary file 2 — Reporting Summary [file 41562_2024_1909_MOESM2_ESM.pdf]

nature portfolio

Corresponding author(s): Caitlin E. Carey and Raymond K. Walters

Last updated by author(s): May 10, 2024

Reporting Summary

Nature Portfolio wishes to improve the reproducibility of the work that we publish. This form provides structure for consistency and transparency in reporting. For further information on Nature Portfolio policies, see our Editorial Policies and the Editorial Policy Checklist.

Statistics

For all statistical analyses, confirm that the following items are present in the figure legend, table legend, main text, or Methods section.

n/a	Confirmed	
☐	☒	The exact sample size (n) for each experimental group/condition, given as a discrete number and unit of measurement
☐	☒	A statement on whether measurements were taken from distinct samples or whether the same sample was measured repeatedly
☐	☒	The statistical test(s) used AND whether they are one- or two-sided<br>*Only common tests should be described solely by name; describe more complex techniques in the Methods section.*
☐	☒	A description of all covariates tested
☐	☒	A description of any assumptions or corrections, such as tests of normality and adjustment for multiple comparisons
☐	☒	A full description of the statistical parameters including central tendency (e.g. means) or other basic estimates (e.g. regression coefficient) AND variation (e.g. standard deviation) or associated estimates of uncertainty (e.g. confidence intervals)
☐	☒	For null hypothesis testing, the test statistic (e.g. F, t, r) with confidence intervals, effect sizes, degrees of freedom and P value noted<br>*Give P values as exact values whenever suitable.*
☐	☒	For Bayesian analysis, information on the choice of priors and Markov chain Monte Carlo settings
☒	☐	For hierarchical and complex designs, identification of the appropriate level for tests and full reporting of outcomes
☐	☒	Estimates of effect sizes (e.g. Cohen's d, Pearson's r), indicating how they were calculated

Our web collection on statistics for biologists contains articles on many of the points above.

Software and code

Policy information about availability of computer code

Data collection	Data consisted of outcomes that were the result of factor analyzing thousands of phenotypes in biobank-scale data, long with genetic data in the form of genotypes. Details on how these factors were extracted and analyzed are available in the manuscript and Methods. Details on how genotypes were obtained in each dataset are available in the Methods section of the manuscript
Data analysis	Data analysis was conducted as specified in the Methods section of the manuscript. Hail Version 2.0 was used for GWAS, polygenic scores were generated with PLINK Version 1.9 and LDpred2. Multiple imputation of core data was performed with MICE (Version 3.16.0), factor analyses and other analyses were performed with relevant packages in R Version 4.2.3, including psych (Version 2.4.1) and lavaan (Version 0.6-3) and relevant packages in Python Version 3.13, including statsmodels (Version 0.13.1), numpy (Version 1.26.3), and lifelines (Version 0.26.4), details of which are provided in the text of the Methods and Supplementary Information. All code has been uploaded and is available at https://github.com/ce-carey/ukb-factor-analysis.

For manuscripts utilizing custom algorithms or software that are central to the research but not yet described in published literature, software must be made available to editors and reviewers. We strongly encourage code deposition in a community repository (e.g. GitHub). See the Nature Portfolio guidelines for submitting code & software for further information.

Data

Policy information about availability of data

All manuscripts must include a data availability statement. This statement should provide the following information, where applicable:

- Accession codes, unique identifiers, or web links for publicly available datasets
- A description of any restrictions on data availability
- For clinical datasets or third party data, please ensure that the statement adheres to our policy

The GWAS results for our 35 factors are available through the GWAS Catalog accession nos. GCST90309336-GCST90309370 (in chronological order of the factors).

Research involving human participants, their data, or biological material

Policy information about studies with human participants or human data. See also policy information about sex, gender (identity/presentation), and sexual orientation and race, ethnicity and racism.

Reporting on sex and gender	Throughout the analyses, we use only sex (i.e., not gender) and are clear throughout that sex was defined based on chromosomes (i.e., XX is "female", XY is "male"). We use chromosomal sex as a covariate in all analyses because we were interested in population-level trends irrespective of sex.
Reporting on race, ethnicity, or other socially relevant groupings	Genetic ancestry was determined using genetic data (using Principal Components analysis). Here we limited our sample to only individuals of European ancestry due to the statistical confounds presented by population stratification, as is standard in the literature. All GWAS and polygenic prediction exercises also controlled for genetic ancestry (the top 20 principal components of the genetic variance-covariance matrix of the genetic data for GWAS analyses and the top 10 for polygenic prediction exercises).
Population characteristics	Those included in this study were participants in the UK Biobank, a longitudinal health study of ~500K volunteers between the ages of 40-69 at recruitment between the years of 2006-2010. See the UK Biobank website (https://www.ukbiobank.ac.uk/), Bycroft et al., 2008 and the sections below for more information.<br><br>In Add Health, the mean birth year of respondents is 1979 (SD = 1.8), and the mean age at the time of assessment (Wave 4) is 29.0 years (SD = 1.8). All phenotypes included in this study come from Wave 4, the latest wave of Add Health data collection (2007-2009).
Recruitment	The UK Biobank (UKB) is a health resource which has the purpose of improving the prevention, diagnosis, and treatment of human disease75. It consists of a prospective cohort of 502,620 men and women aged 40-69 recruited in the years 2006-2010 throughout the United Kingdom. The touchscreen questionnaire is a collection of self-reported information regarding general health, dietary habits, physical activity, psychological and cognitive states, sociodemographic factors, etc. We began with 361,194 unrelated individuals of European genetic ancestry who passed quality control measures (https://www.nealelab.is/uk-biobank/ukbround2announcement).<br><br>Add Health originated as an in-school survey of a nationally representative sample of US adolescents enrolled in grades 7 through 12 during the 1994-1995 school year. Respondents were born between 1974 and 1983, and a subset of the original Add Health respondents has been followed up with in-home interviews, which allows researchers to assess correlates of outcomes in the transition to early adulthood.<br><br>No
Ethics oversight	Use of the UK Biobank data was approved under application 31063. Analysis of the UK Biobank data was reviewed by the Partners HealthCare IRB (Partners Human Research), which determined in expedited review that the project met the US federal criteria definition of “not human subjects research.”<br><br>Analysis of the Add Health data was reviewed by the Office of Research Subject Protection (OSRP) at the Broad Institute of MIT and Harvard, which determined that the project met US federal criteria for exemption from IRB review (Project #0001 titled “Genetic and environmental factors influencing complex social behavior”).<br><br>Informed consent was obtained by participants who chose to participate in both the UK Biobank and Add Health studies, and this consent was handled by the teams curating those datasets.

Note that full information on the approval of the study protocol must also be provided in the manuscript.

Field-specific reporting

Please select the one below that is the best fit for your research. If you are not sure, read the appropriate sections before making your selection.

☐ Life sciences ☒ Behavioural & social sciences ☐ Ecological, evolutionary & environmental sciences

For a reference copy of the document with all sections, see nature.com/documents/nr-reporting-summary-flat.pdf

Behavioural & social sciences study design

All studies must disclose on these points even when the disclosure is negative.

Study description	This study used data from the UK Biobank and attempted to better characterize the measured data using dimensionality reduction methods, most specifically factor analysis. Briefly, we performed factor analyses on the phenotypes in the UK Biobank, extracted 35 orthogonal factors using these methods, and then ran follow-up correlational analyses, including genome-wide association studies and polygenic scoring, of our factors. All data are quantitative.
Research sample	Using the UK Biobank, we examined 2,772 phenotypes in unrelated individuals with predominantly estimated European genetic ancestry (N=361,144). We performed replication exercises using polygenic scores for 3,414 individuals in the Add Health study. We chose these samples because of the large and required sample size for genetic analyses (UK Biobank) and for existence of well-phenotyped replication data (Add Health). the UK Biobank is not a nationally representative study, while the Add Health study is a US-based nationally representative study.
Sampling strategy	Analytic samples were decided by using the samples of subsamples that had the largest N for a given outcome under study. This strategy was chosen, because large samples are prioritized to have enough statistical power to isolate small genetic associations. This is common practice in the field.
Data collection	The UK Biobank (UKB) is a health resource which has the purpose of improving the prevention, diagnosis, and treatment of human disease75. It consists of a prospective cohort of 502,620 men and women aged 40-69 recruited in the years 2006-2010 throughout the United Kingdom. The touchscreen questionnaire is a collection of self-reported information regarding general health, dietary habits, physical activity, psychological and cognitive states, sociodemographic factors, etc. We began with 361,194 unrelated individuals of European genetic ancestry who passed quality control measures (https://www.nealelab.is/uk-biobank/ukbround2announcement).<br><br>Add Health originated as an in-school survey of a nationally representative sample of US adolescents enrolled in grades 7 through 12 during the 1994-1995 school year. Respondents were born between 1974 and 1983, and a subset of the original Add Health respondents has been followed up with in-home interviews, which allows researchers to assess correlates of outcomes in the transition to early adulthood. In Add Health, the mean birth year of respondents is 1979 (SD = 1.8), and the mean age at the time of assessment (Wave 4) is 29.0 years (SD = 1.8). All phenotypes included in this study come from Wave 4, the latest wave of Add Health data collection (2007-2009).
Timing	The UK Biobank is a prospective cohort of 502,620 men and women aged 40-69 recruited in the years 2006-2010 throughout the United Kingdom.<br><br>Add Health originated as an in-school survey of a nationally representative sample of US adolescents enrolled in grades 7 through 12 during the 1994-1995 school year. Respondents were born between 1974 and 1983, and a subset of the original Add Health respondents has been followed up with in-home interviews, which allows researchers to assess correlates of outcomes in the transition to early adulthood.
Data exclusions	We use only individuals of European ancestry due to the statistical confounds presented by population stratification, as is standard in the literature. Individuals were differentially included/excluded at various steps of the analysis due to data availability; this is extensively documents in the Methods and Supplementary Information.
Non-participation	Participants were allowed to drop out of the study, or to not respond to any individual survey items, at any point, for both the UK Biobank and Add Health samples. We considered missingness extensively in the development of our analytic methods and provide details with the manuscript. However, we can of course account for all bias due to non-participation and/or ascertainment.
Randomization	No randomization was performed; the study was exploratory/cross-sectional/observational.

Reporting for specific materials, systems and methods

We require information from authors about some types of materials, experimental systems and methods used in many studies. Here, indicate whether each material, system or method listed is relevant to your study. If you are not sure if a list item applies to your research, read the appropriate section before selecting a response.

Materials & experimental systems

n/a	Involved in the study
☒	☐ Antibodies
☒	☐ Eukaryotic cell lines
☒	☐ Palaeontology and archaeology
☒	☐ Animals and other organisms
☒	☐ Clinical data
☒	☐ Dual use research of concern
☒	☐ Plants

Methods

n/a	Involved in the study
☒	☐ ChIP-seq
☒	☐ Flow cytometry
☒	☐ MRI-based neuroimaging

Plants

Seed stocks	n/a
Novel plant genotypes	n/a
Authentication	n/a
